# Nanoscale Disentangling of Strain and Doping in Monolayer Graphene Grown on Cu-Based Growth Template and Transferred onto Si Substrate by Combining TERS, Raman Correlation Analysis, and PCA

**DOI:** 10.1021/acsomega.6c05883

**Published:** 2026-08-06

**Authors:** Anacleto Proietti, Luca Buccini, Ginevra Rossetti, Livia Angeloni, Pierfrancesco Atanasio, Simone Bandini, Isabella Chiarotto, Roberto Colarelli, Federico Marini, Francesco Mura, Daniele Passeri, Gianluca Sbardella, Elena Tomaselli, Angelica Accorinti, Corrado Di Conzo, Alba Centeno, Amaia Zurutuza, Marco Rossi

**Affiliations:** † Department of Basic and Applied Sciences for Engineering, 9311 Sapienza University of Rome, Via A. Scarpa 16, 00161 Rome, Italy; ‡ Department of Chemistry, Sapienza University of Rome, Piazzale Aldo Moro 5, 00185 Rome, Italy; § Research Center for Nanotechnology Applied to Engineering of Sapienza University of Rome, Piazzale Aldo Moro 5, 00185 Rome, Italy; ∥ Department of Applied Science and Technology (DISAT), Polytechnic University of Turin, Corso Castelfidardo 39, 10129 Turin, Italy; ⊥ 383835 Graphenea S.A, Paseo Mikeletegi 83, 20009 San Sebastián, Spain

## Abstract

Understanding and decoupling the effects of strain and doping in graphene remains a central challenge in Raman spectroscopy, as far-field measurements inherently average local variations. In this work, we combine conventional Raman spectroscopy, tip-enhanced Raman spectroscopy (TERS), G–2D bands correlation analysis, and principal component analysis (PCA) to investigate graphene grown on copper (Cu) and transferred onto silicon (Si) substrates, with the aim of probing substrate-dependent spectral variability across different length scales. Far-field Raman measurements show distinct but relatively compact spectral distributions for graphene on Cu and Si, with trends that can be qualitatively associated with mixed strain- and dopinglike contributions. These measurements primarily reflect a spatially averaged response, which masks local variations. In contrast, TERS reveals a significantly broader and spatially heterogeneous spectral behavior, particularly for graphene on Cu, whereas graphene on Si exhibits a more compact distribution. These differences indicate that substrate-dependent interactions and transfer processes influence not only the average spectral response but also its local variability. Correlation analysis based on G–2D trends provides a qualitative framework to identify dominant spectral tendencies, while PCA enables a data-driven description of spectral variability without relying on peak fitting. PCA distinguishes the main substrate-dependent contribution from additional sources of variability and, through spatial mapping of principal components, reveals that these variations are spatially structured at the nanoscale in TERS measurements, whereas Raman maps remain comparatively smooth. Overall, the combined approach demonstrates that TERS does not simply enhance the Raman signal locally but provides access to the distribution of local spectral variations that are averaged out in far-field measurements. This methodology offers a robust framework for the nanoscale investigation of graphene and can be extended to other two-dimensional materials, where local heterogeneity plays a key role in determining macroscopic properties.

## Graphical abstract

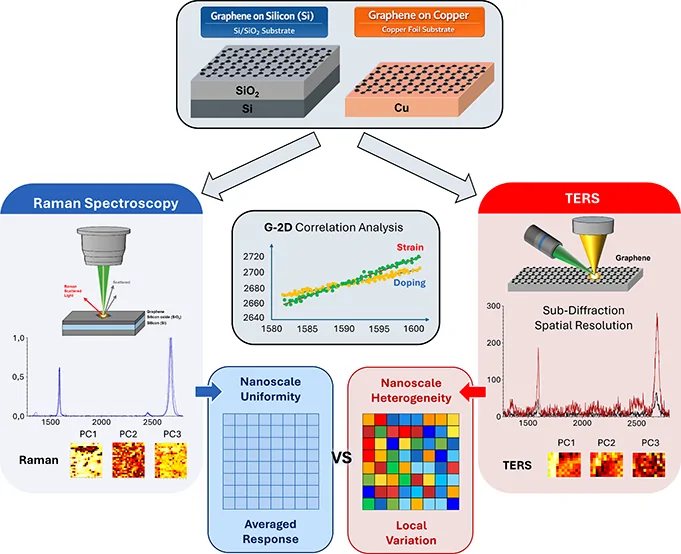

## 1 Introduction

Graphene has attracted widespread attention due to its outstanding electrical and mechanical properties as well as its unique optoelectronic response, making it a key material for applications in nanoelectronics, sensing, and energy devices.^1,2^ However, its performance is highly sensitive to local variations in strain and doping, which can strongly affect both its electronic band structure and vibrational response.^3–5^ The ability to distinguish and interpret these contributions is, therefore, crucial for both fundamental studies and technological implementation.

Raman spectroscopy is one of the most established and powerful techniques for graphene and 2D material characterization, providing insights into structural quality, defects, and electronic properties.^6–9^ In particular, the position of the G and 2D bands is strongly influenced by both strain and doping,^10^ and their correlation has been widely used as a framework to infer the dominant physical contributions.^11^ Nevertheless, conventional far-field Raman spectroscopy is inherently limited by diffraction, resulting in a spatial resolution of several hundred nanometers and, therefore, an average response over heterogeneous regions.

This limitation is especially relevant for graphene grown on copper and subsequently transferred to silicon substrates. In these systems, local variations in morphology, substrate interaction, and transfer-induced defects can introduce significant nanoscale heterogeneity, affecting both the strain distribution and charge carrier concentration.^12–14^ Therefore, far-field Raman measurements may obscure the presence of distinct local spectral regimes, complicating the interpretation of the strain and doping effects.

Tip-enhanced Raman spectroscopy (TERS) overcomes these limitations by combining scanning probe microscopy with localized plasmonic enhancement, enabling Raman measurements with spatial resolution down to the nanometer scale.^15–20^ By confining the electromagnetic field at the apex of a metallic tip, TERS provides access to local vibrational information that is otherwise averaged out in conventional Raman spectroscopy, while also introducing additional near-field effects related to tip–sample interactions. Recent developments in TERS probe design, including the use of alternative materials such as Titanium Nitride (TiN), have further improved the robustness and applicability of the technique, particularly in environments compatible with semiconductor processing.^21,22^ In addition, established experimental configurations have demonstrated the capability of TERS to probe complex nanoscale systems with high sensitivity.^23^ In the context of graphene and other nanostructured materials, TERS has shown strong potential for revealing local heterogeneity associated with strain, doping, and defects.^24–27^ However, the interpretation of TERS data in terms of these physical contributions remains challenging. While correlation analysis of G and 2D peak positions provides a useful framework, the coexistence of multiple local effects and the complexity of near-field interactions can limit a straightforward quantitative interpretation.^10,28^ This reflects a broader challenge in multiscale characterization approaches, where combining complementary techniques is necessary to properly interpret complex material systems.^29^

To address these challenges, data-driven approaches such as PCA have been increasingly employed to identify underlying spectral patterns and separate distinct contributions without relying on explicit peak fitting or predefined models.^30–32^ Such methods can be particularly valuable when dealing with large spectral data sets and heterogeneous systems.

In this work, we combine conventional Raman spectroscopy, TERS, G–2D correlation (slope) analysis, and PCA to investigate monolayer graphene grown on copper and monolayer graphene transferred onto silicon substrates. Here, we investigate substrate-dependent nanoscale heterogeneity to disentangle strain- and doping-related spectral tendencies. By directly comparing far-field and near-field measurements, we show that TERS provides access to local spectral regimes that are not resolved in conventional Raman spectroscopy. The combined use of correlation analysis and PCA further supports the identification of distinct spectral populations, providing a robust framework for the nanoscale characterization of graphene and of two-dimensional materials.

## 2 Materials and Methods

### 2.1 Sample Preparation

Monolayer graphene samples were grown by chemical vapor deposition (CVD) on commercially purchased polycrystalline copper foil and subsequently transferred onto Si/SiO_2_ substrates using a standard semidry transfer process (Figure 1). Before graphene growth, the Cu foil was annealed at high temperature under an inert atmosphere. The Si substrates consisted of Si wafers covered with a thermally grown 90 *nm* SiO_2_ layer supplied by an external wafer provider. Prior to graphene transfer, the Si/SiO_2_ surface was activated by an ozone treatment.

**Figure 1:**
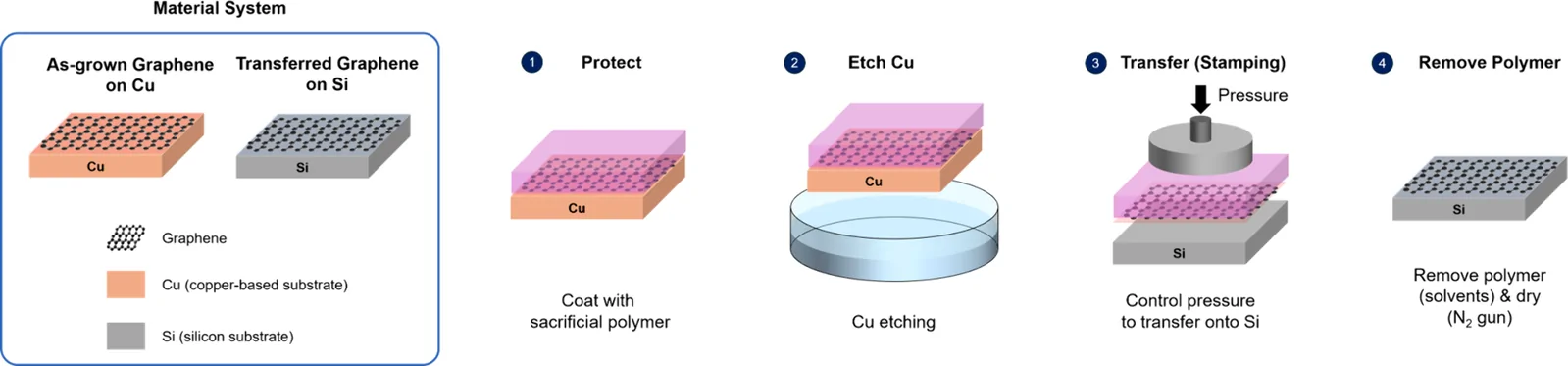
Schematic illustration of the monolayer graphene systems investigated in this work and of the semidry transfer process from Cu-based growth substrates to Si substrates. The scheme summarizes sacrificial polymer protection, Cu etching, pressure-assisted transfer onto Si, and final polymer removal.

The semidry transfer process consisted of protecting the graphene grown on Cu with a sacrificial polymer layer to withstand the chemical etching of the Cu substrate. The polymer/graphene stack was then transferred onto the Si/SiO_2_ substrate by controlling the pressure during the stamping. Finally, the sacrificial polymer was removed by solvent cleaning followed by drying with a nitrogen gun. Both as-grown graphene on polycrystalline Cu and transferred graphene on Si/SiO_2_ were investigated to evaluate the influence of the substrate and the transfer procedure on the local structural and electronic properties of graphene.

### 2.2 Raman Spectroscopy

Conventional Raman measurements were performed using a confocal Raman microscope (Renishaw inVia). A 532 *nm* excitation laser was employed in combination with a 100× objective (NA = 0.85), providing diffraction-limited spatial resolution. The laser power was maintained at approximately 5 mW on the sample to minimize local heating effects. For graphene transferred onto Si, spectra were acquired using an integration time of 1 *s* with 5 accumulations. Raman maps were collected over a 34 × 15-point grid with a pixel size of 1 *μm* × 1 *μm*. For graphene on Cu, longer acquisition conditions of 10 s with 10 accumulations were adopted to compensate for the lower Raman response of graphene on the metallic substrate. Raman maps were collected over a 22 × 20-point grid with a pixel size of 1 *μm* × 1 *μm*.

Spectra were acquired in the spectral regions corresponding to the G (∼1580–1600 *cm* ^*–1*^) and 2D (∼2600–2700 *cm* ^*–1*^) bands of graphene. Spectral preprocessing, including baseline correction and normalization, was performed using WiRE 5.4 software. After baseline correction, each Raman/TERS spectrum was normalized by setting the maximum spectral intensity to 1; for all spectra reported in the figures, this maximum corresponded to the graphene 2D band. The positions of the G and 2D Raman bands were extracted after baseline correction by fitting each band with a mixed Gaussian–Lorentzian profile using WiRE 5.4 software. The fitted peak center was used as the Raman peak position for the subsequent G-2D correlation analysis. No smoothing was applied prior to fitting, to avoid artificial modification of the spectral line shape.

### 2.3 Tip-Enhanced Raman Spectroscopy (TERS)

TERS measurements were carried out by coupling the Renishaw inVia Raman system to a Bruker Innova atomic force microscope. Measurements were performed in tapping mode using TiN-coated TERS probes (ScanSens), designed for improved mechanical stability and compatibility with clean-room environments. The experimental configuration follows established approaches for TERS measurements in nanoscale systems.^23^

A 532 nm excitation wavelength was used to ensure direct comparability with far-field Raman measurements. The laser power was maintained at approximately 5 *mW* on the sample for both substrates, consistent with the conventional Raman measurements. Spectra were acquired in both tip-in (near-field enhanced) and tip-out (far-field reference) configurations. The remaining acquisition parameters were optimized independently for the two samples because they exhibited different stability during TERS mapping. For graphene on Cu, TERS maps were acquired using 10 *s* integration time, 1 accumulation, and a 12 × 9-point grid with a pixel size of 275 *nm* × 275 *nm*. For graphene transferred onto Si/SiO_2_, TERS maps were acquired using 2 *s* integration time, 10 accumulations, and a 13 × 10-point grid with a pixel size of 75 *nm* × 75 *nm*. These conditions were selected to obtain stable and reproducible spectra while minimizing the effects of sample drift during mapping. The near-field enhancement enables spatial resolution in the order of tens of nanometers, allowing the investigation of local spectral variations in graphene.

### 2.4 SEM and EDX Analysis

Scanning electron microscopy (SEM) was performed using a field emission SEM (Zeiss Auriga) equipped with an energy-dispersive X-ray spectroscopy (EDX) system fitted with an Oxford Instruments Ultim Max 65 *mm* ^2^ silicon drift detector (SDD).

SEM imaging was used to assess the surface morphology and continuity of the graphene layers, while EDX provided complementary compositional information. These analyses were used as supporting characterizations to contextualize the spectroscopic results.

### 2.5 Atomic Force Microscopy (AFM)

AFM measurements were performed using a Bruker Dimension Icon system. Topographic imaging was carried out in tapping mode using HA_HR/50 probes (ScanSens). Images were acquired over areas of 5 *μm* × 5 *μm* with a resolution of 512 points per line. AFM data were processed using Gwyddion software. A plane leveling procedure was applied to remove background tilt and sample inclination, ensuring an accurate representation of the surface topography. The processed images were used to evaluate the morphological homogeneity of graphene on both Cu and Si substrates, providing complementary information to the spectroscopic analysis.

### 2.6 Correlation (Slope) Analysis of Raman Bands

The positions of the G and 2D Raman bands were extracted from each spectrum and used to construct G-2D correlation plots. This analysis relies on the fact that strain and charge doping affect the G and 2D phonon frequencies in different ways. Strain modifies the C–C bond length and therefore shifts both the G and 2D bands, with the 2D band showing a stronger sensitivity because of its larger Grüneisen parameter and double-resonance origin. In contrast, charge doping mainly affects the G band through nonadiabatic electron–phonon coupling and phonon stiffening, while the 2D band exhibits a weaker and more complex dependence on carrier concentration. Therefore, strain-dominated and doping-dominated variations follow different trajectories in the ω_2D_ versus ω_G_ correlation plot. The experimental data were therefore compared with reference slopes previously reported for strain- and doping-related trends in graphene.^19,20^ This approach was used to identify the dominant spectral tendency in each region, rather than to provide an absolute quantitative separation of strain and doping contributions.

Linear fits to the experimental data sets were compared with theoretical reference lines corresponding to pure strain and pure doping conditions. This approach allows for the identification of dominant spectral tendencies rather than an absolute quantification of strain and doping.

### 2.7 Principal Component Analysis (PCA)

PCA was performed using an in-house written function running under MATLAB (R2025b, The Mathworks, Natick, MA) on both Raman and TERS spectral data sets. Prior to PCA, Raman/TERS spectra were baseline-corrected in WiRE 5.4 and restricted to the spectral regions of the graphene G and 2D bands, namely, 1300–1700 cm^–1^ and 2400–2800 *cm* ^*–1*^, respectively. For each individual spectrum, the G and 2D bands were independently normalized by setting the maximum intensity of each band to 1 before PCA. This preprocessing was applied to minimize the influence of absolute and relative intensity variations, which can be affected by experimental factors such as local enhancement efficiency, tip–sample coupling, focusing conditions, surface morphology, and local optical response. In this way, PCA was mainly driven by changes in band position, width, and spectral shape, which are more directly related to strain and doping effects in graphene.

PCA was employed as an unsupervised statistical method to identify the main sources of spectral variance and to highlight clustering of spectra associated with different substrates and measurement configurations (Raman vs TERS). This approach enables the interpretation of spectral variations associated with strain and doping without requiring explicit peak-fitting procedures.

## 3 Results and Discussion

### 3.1 Morphological Characterization

The graphene samples were first characterized from a morphological and compositional standpoint to provide a structural framework for subsequent spectroscopic analysis. Representative SEM, AFM, and EDX results for graphene on Cu and graphene transferred onto Si are shown in Figure 2.

**Figure 2:**
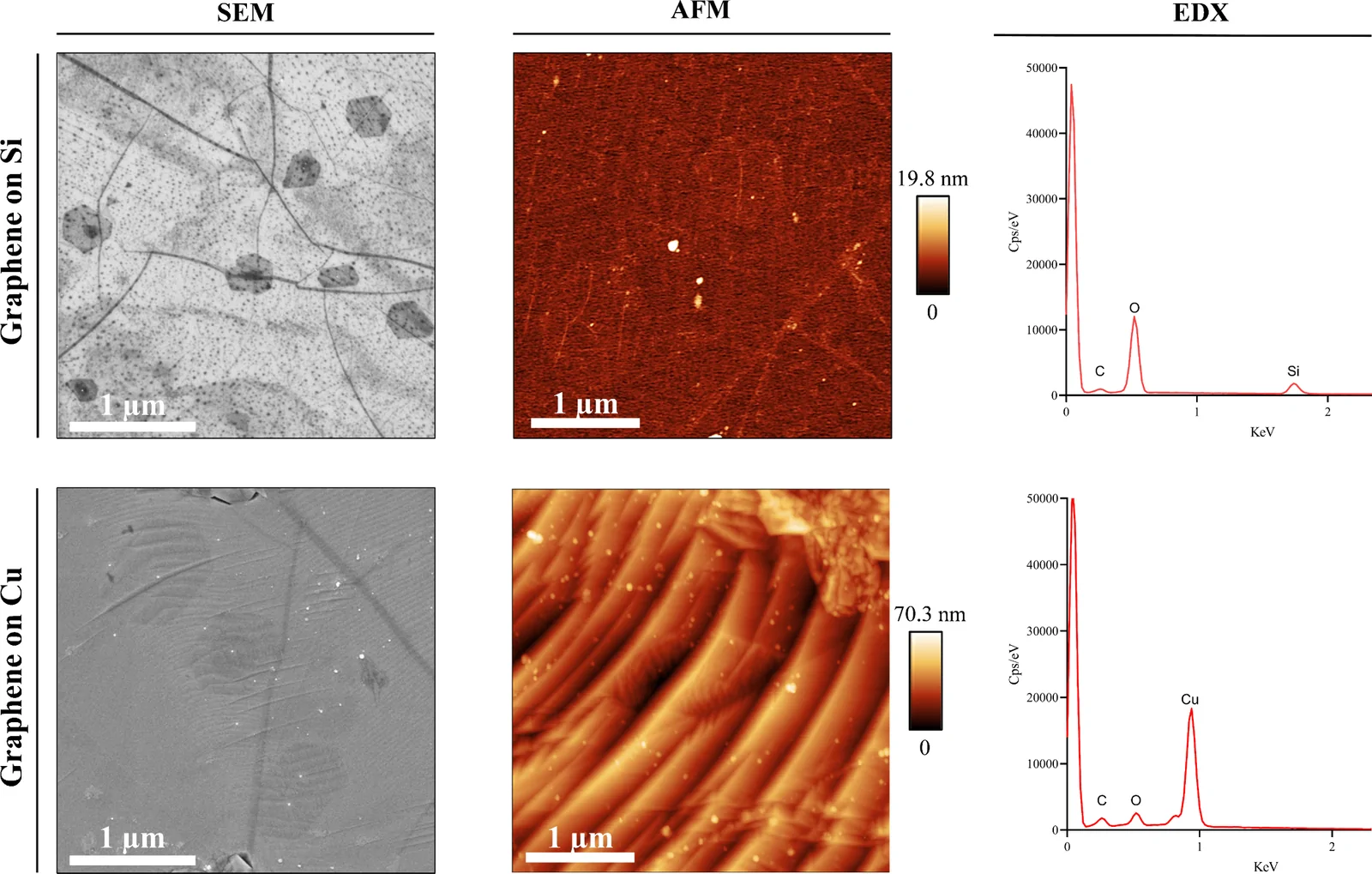
Graphene on Si (top row) and graphene on Cu (bottom row). For each sample, the first column shows representative SEM micrographs. The second column reports AFM topography maps (orange scale). The third column presents EDX spectra acquired from the investigated regions.

SEM imaging of graphene on Si reveals a relatively uniform and continuous coverage with limited microscale inhomogeneities. AFM topography confirms a smoother surface morphology, although small features attributable to the transfer process, such as wrinkles or residual contamination, can still be observed locally. In contrast, graphene on Cu exhibits a more irregular morphology, as highlighted by SEM images showing substrate-related features and growth-induced inhomogeneities. AFM measurements further evidence a higher degree of surface corrugation and roughness on the Cu sample, consistent with the influence of the metallic substrate and the intrinsic characteristics of CVD growth on Cu.

EDX analysis shows the expected elemental composition of the investigated samples, with signals mainly associated with the underlying substrates, namely, Si and Cu. The carbon signal is consistent with the presence of graphene on the surface; however, considering the limited sensitivity and spatial resolution of EDX for atomically thin carbon layers, this analysis was used only as a qualitative check of the sample composition and of the absence of major unexpected contaminants. The oxygen signal observed in the spectra was also considered. For graphene transferred onto Si, the relatively stronger oxygen contribution can be attributed to the SiO_2_ layer present on the Si substrate. For graphene grown on Cu, the weaker oxygen signal can be reasonably associated with surface oxidation of the copper substrate and/or adsorbed oxygen-containing species. These oxygen-containing interfacial layers or surface species may contribute to local charge transfer and substrate-induced interactions and therefore can play a role in the strain and doping effects discussed in Raman analysis. No conductive coating was applied prior to SEM imaging so that the observed morphology reflects the native surface.

Overall, while continuous graphene coverage is observed in both cases, graphene on Cu shows a higher degree of local heterogeneity compared with graphene on Si, which is expected to impact the local strain and doping distributions addressed in the following sections.

### 3.2 Raman Spectroscopy: Substrate-Dependent Baseline Response

The Raman response of graphene on Si and Cu was first analyzed to establish a far-field reference for the interpretation of local spectroscopic variations. Representative spectra for both systems are shown in Figure 3, where multiple acquisitions per sample (solid, dashed, and dotted curves) highlight spectral variability.

**Figure 3:**
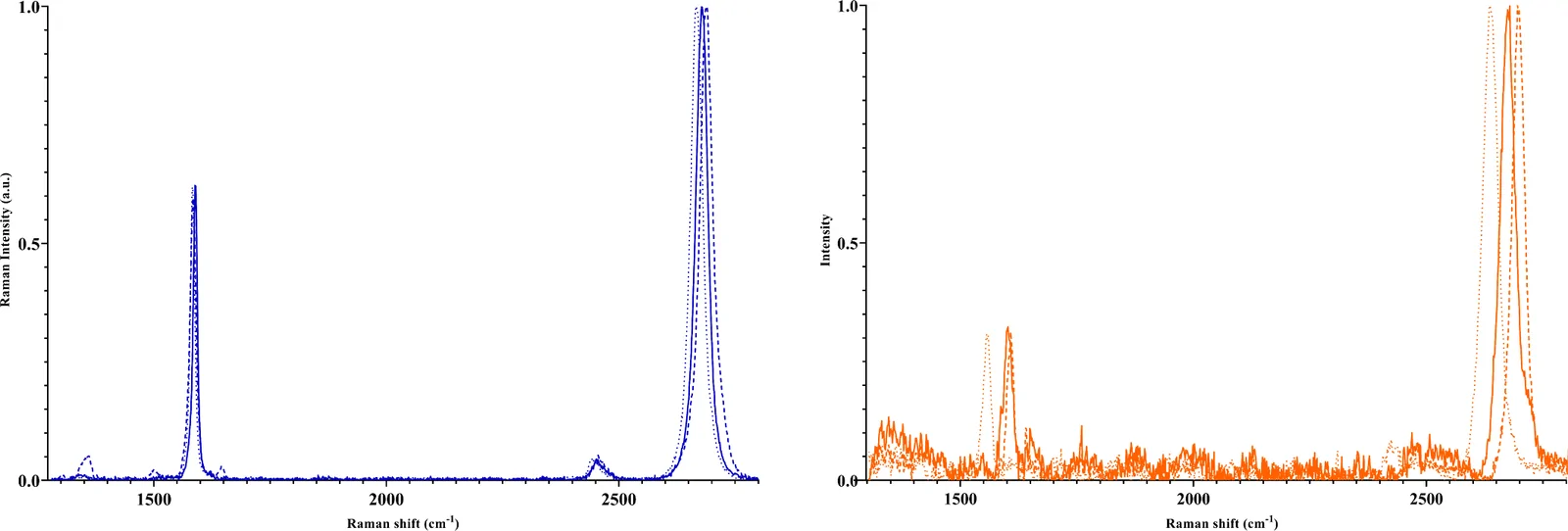
Raman spectra of graphene on Si (blue, left) and graphene on Cu (orange, right). For each sample, three spectra (solid, dashed, and dotted) are reported with visible shifts in the G-band and 2D band positions across different measurement points.

Both systems exhibit the characteristic Raman features of graphene. The G band, observed in the range of ∼ 1580–1600 *cm* ^*–1*^, originates from the first-order Raman scattering of the in-plane E_2g_ optical phonon at the Brillouin-zone center. The 2D band, located around ∼ 2600–2700 *cm* ^*–1*^, is the second-order overtone of the defect-activated D band and arises from a double-resonance Raman process involving transverse optical phonons near the K point. The presence of a well-defined 2D band, together with the G band, confirms the presence of high-quality graphene layers. However, as visible in Figure 3, graphene on Cu shows a more pronounced dispersion in the position of both G and 2D bands across different spectra, whereas graphene on Si displays more limited peak shifts and a more consistent spectral profile.

In addition, the Raman signal of graphene on Cu appears significantly noisier, with a lower signal-to-noise ratio compared to graphene on Si, as shown by the comparison between the two far-field signals reported in Figure S1 of the Supporting Information. This effect is mainly attributed to the metallic nature of the Cu substrate, which can introduce a stronger background contribution, enhanced reflectivity, absorption losses, and damping of graphene Raman contrast. These effects can reduce the effective Raman visibility of graphene and increase the apparent spectral noise. In contrast, for graphene transferred onto Si, the presence of the oxide/SiO_2_ layer can influence the Raman response through multiple reflections and optical interference effects, as commonly reported for graphene on dielectric-layer/substrate systems. Therefore, the different optical responses of the two substrates, including metallic background and damping effects for Cu and interference-related intensity modulation for Si/SiO_2_, may contribute to the observed differences in Raman signal quality, while the strain- and doping-related analysis remains based primarily on peak positions and normalized spectral features rather than on absolute Raman intensity.

These differences indicate a higher degree of variability in the local spectroscopic response for graphene on Cu compared to the more homogeneous behavior observed on Si. This trend is consistent with the morphological observations discussed in section 3.1, where graphene on Cu exhibited increased surface irregularity.

To further investigate these variations, we analyzed the correlation between the G and 2D peak positions (Figure 4).

**Figure 4:**
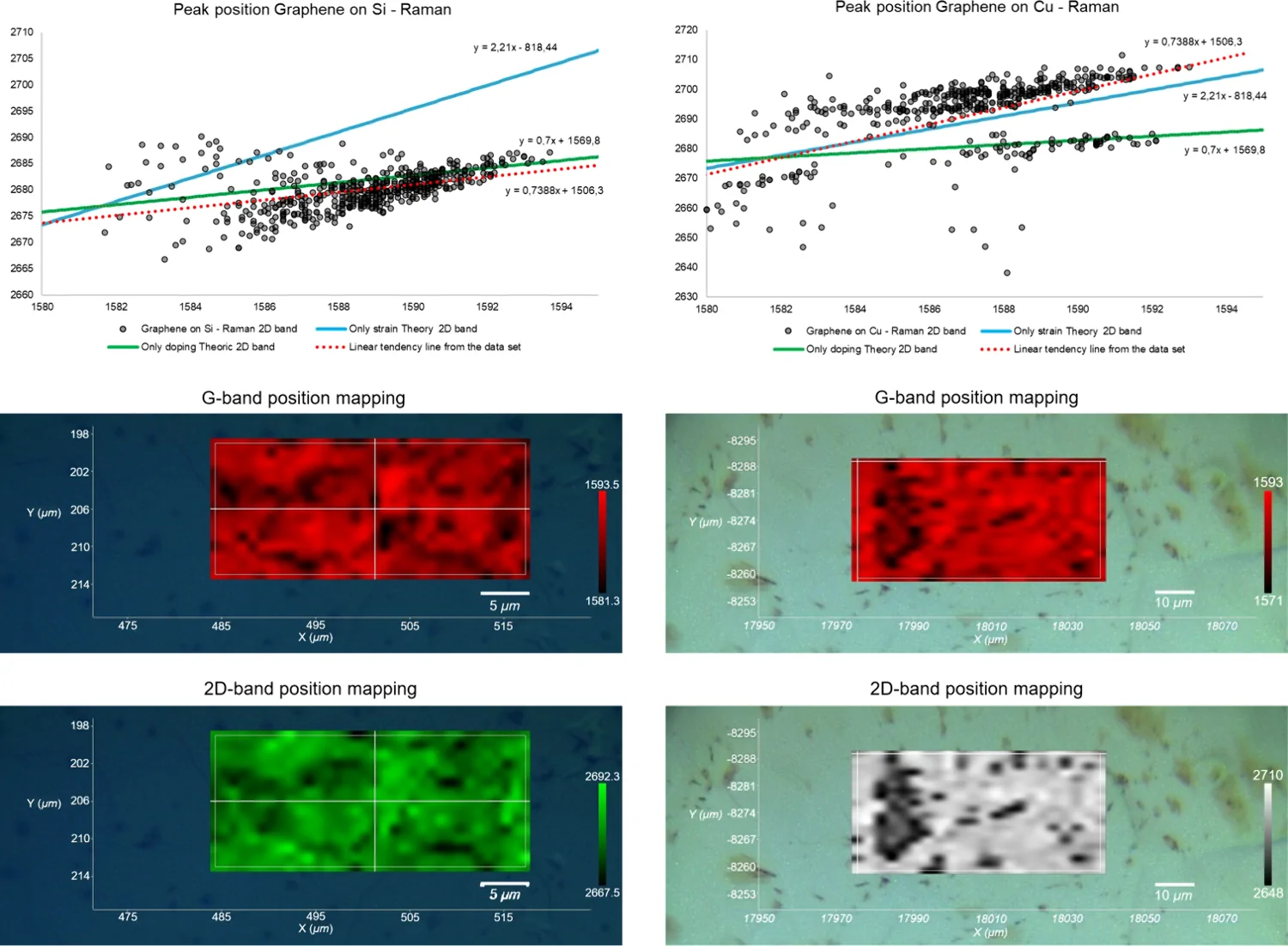
Correlation between G and 2D peak positions for graphene on Si (left column) and graphene on Cu (right column), together with spatial maps of the fitted peak positions. Top panels: scatter plots of the experimental data (black dots) compared with reference trends for strain-dominated (blue line) and doping-dominated (green line) regimes, along with the linear fit of the data sets (red dotted line). Middle panels: spatial distribution of the G band position extracted from peak fitting. Bottom panels: spatial distribution of the 2D band position extracted from peak fitting. The graphene on Si data set consists of 510 spectra acquired over a 34 × 15-point grid with 1 μm step size in both x and y directions. The graphene on Cu data set consists of 440 spectra acquired over a 22 × 20-point grid with 1 μm step size in both x and y directions.

In both systems, the data follow a quasi-linear trend, which can be qualitatively compared with reference slopes associated with strain- and doping-dominated regimes.^19,20^ Graphene on Cu shows a broader distribution of points with a tendency to lie between the strain and doping reference lines, suggesting the coexistence of multiple contributions. In contrast, graphene on Si exhibits a more compact distribution, closer to doping-like behavior.

It is important to note that although correlation analysis provides a useful framework to identify dominant spectral tendencies, the far-field nature of Raman spectroscopy inherently averages the response over regions that may include multiple local environments. Therefore, the observed trends should be interpreted as effective contributions rather than direct measurements of the local strain or doping. Overall, while conventional Raman spectroscopy captures clear substrate-dependent differences, it does not directly resolve the nanoscale heterogeneity that underlies these variations. This limitation motivates the use of TERS, as discussed in the following section, to probe local spectral features beyond the diffraction limit.

### 3.3 Tip-Enhanced Raman Spectroscopy: Nanoscale Spectral Heterogeneity

To investigate the local spectroscopic response beyond the spatial averaging inherent to far-field Raman measurements, TERS analyses were performed on both graphene on Si and graphene on Cu. Representative spectra and the corresponding G–2D correlation plots are reported in Figures 5 and 6.

**Figure 5:**
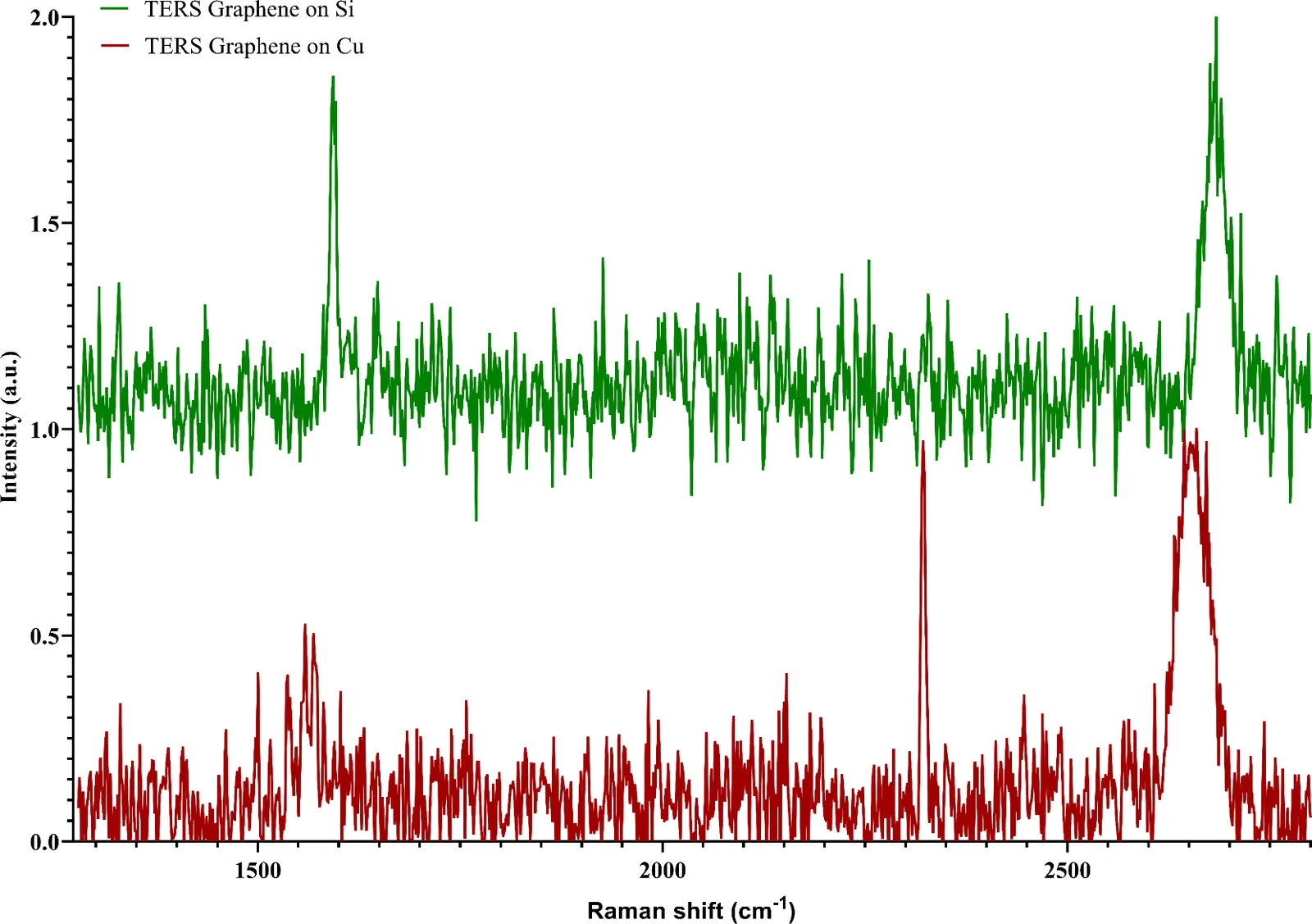
TERS spectra acquired on graphene on Si (green) and graphene on Cu (red), highlighting differences in near-field Raman response between the two substrates. The enhanced spectral features reflect local signal amplification at the tip–sample junction. A peak around ∼ 2320 cm^–1^ is attributed to molecular N_2_ from the ambient environment. Tip-in and tip-out comparisons are reported in Figures S2 and S3 (Supporting Information) together with a comparison with the far-field in Figures S4 and S5 (Supporting Information).

**Figure 6:**
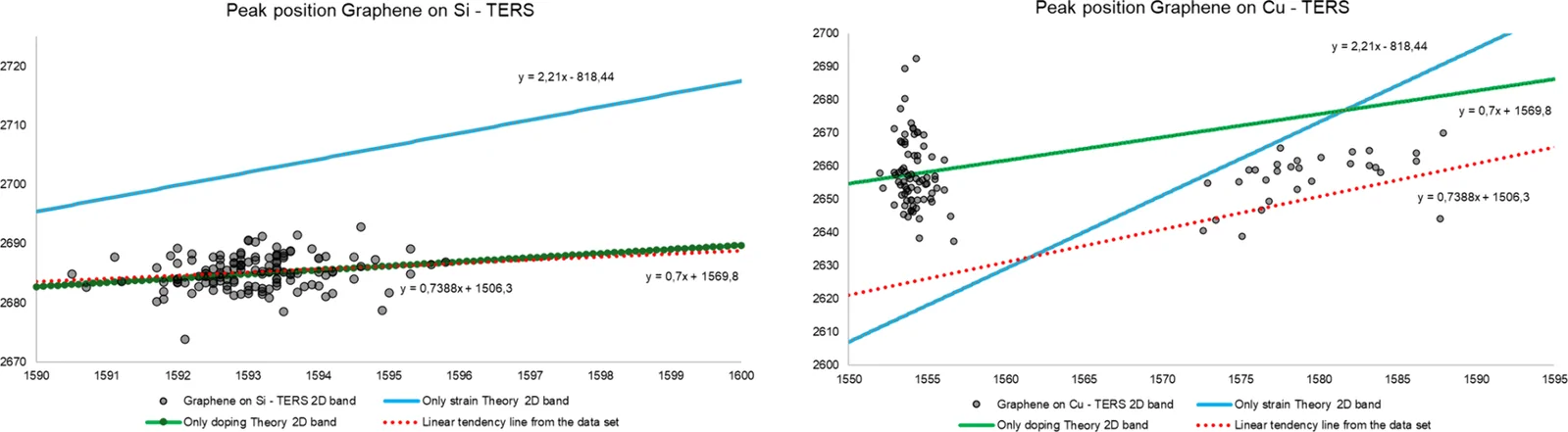
Correlation between G and 2D peak positions from TERS measurements for graphene on Si (left) and graphene on Cu (right). Experimental data (black dots) are compared with reference trends for strain-dominated (blue line) and doping-dominated (green line) regimes, together with the linear fit of the data sets (red dotted line).

The spectra shown in Figure 5 and in the Supporting Information were baseline-corrected and normalized using the same procedure; no smoothing or averaging was applied. The apparent noise level may vary locally across the TERS maps because of changes in tip–sample coupling, local surface morphology, near-field enhancement efficiency, and substrate-dependent background contributions. The additional peak observed around ∼ 2320 *cm* ^*–1*^ in the graphene-on-Cu TERS spectra (Figure 5) is assigned to molecular N_2_, likely enhanced by the local tip–sample environment and by the metallic substrate. This feature is not associated with graphene vibrational modes and was not considered in the strain/doping analysis or in the PCA, which focused on the G and 2D spectral regions. TERS measurements confirm the effectiveness of the near-field coupling, with a clear enhancement of the Raman signal under tip-in conditions (see Figures S2 and S3). In addition, a peak around ∼ 2320 *cm* ^*–1*^, attributed to molecular N_2_, is observed only in the case of graphene on Cu, suggesting a contribution related to the local tip–sample environment and possibly enhanced by the metallic substrate.

For graphene on Si, the G–2D correlation plot obtained from TERS data shows a compact and homogeneous distribution. The data points are closely clustered and follow a consistent trend, which appears to be aligned with a predominantly doping-like behavior. This indicates that, at the nanoscale, graphene on Si exhibits a relatively uniform local spectroscopic response, with limited variability across the probed regions.

In contrast, graphene on Cu exhibits a markedly broader and nonuniform distribution in the G–2D correlation plot. As visible in Figure 6, the data do not follow a single linear trend but instead show a scattered and partially clustered behavior, with distinct groups of points separated in the G–2D space. This suggests the presence of multiple local spectral regimes, indicating strong nanoscale heterogeneity. The distribution also deviates from both the pure strain and pure doping reference lines, highlighting a complex interplay of local effects that cannot be reduced to a single dominant contribution.

A direct comparison between the Raman and TERS results highlights a key aspect of the near-field approach. While far-field Raman measurements provide an average description of the system, TERS reveals local spectral variations that would otherwise be hidden. In particular, the complex and structured distribution observed for graphene on Cu is not evident in the Raman data, demonstrating that far-field measurements mask significant nanoscale variability.

It is important to note that the interpretation of TERS data in terms of strain and doping remains qualitative as the coexistence of multiple local effects and the complexity of the near-field interaction prevent a straightforward quantitative decoupling. Nevertheless, the clear differences between Cu and Si substrates indicate that the underlying physical contributions are not uniformly distributed on the nanoscale.

Overall, these results demonstrate that TERS provides access to local spectroscopic information beyond the diffraction limit, enabling the identification of substrate-dependent nanoscale heterogeneity in graphene and offering deeper insights into the role of growth and transfer processes.

### 3.4 Principal Component Analysis: Data-Driven Identification of Spectral Regimes

To disentangle substrate-dependent effects from intrinsic spectral variability, PCA was performed at two different levels: separately on each data set (graphene on Cu and graphene on Si) and on the combined data set including both substrates. In this framework, each principal component (PC) represents a linear combination of spectral features, while the corresponding score maps describe the spatial distribution of these spectral variations.

#### 3.4.1 Raman Analysis

PCA applied independently to Raman spectra acquired on graphene on Cu and graphene on Si reveals a clear clustering on Cu and no clustering on Si (see Supplementary Figures S6–S7). In both cases, the variance is mainly distributed along PC1, but only for the Cu sample some clustering became visible. The corresponding loading plots indicate that the variability is primarily associated with fluctuations in the G and 2D bands, suggesting that intradata set variance arises from local variations in strain, doping, or measurement conditions rather than from distinct spectral populations. These results indicate that, within each substrate, Raman spectroscopy probes relatively homogeneous spectral features, although smaller-scale variations cannot be excluded.

When PCA is performed on the combined Raman data set, a clear separation between graphene on Cu and Si emerges mostly along PC1, with a further contribution on PC2, which accounts for additional within-class heterogeneity, particularly reflecting the broader dispersion of graphene-on-Cu samples, as shown in Figure 7. This indicates that the dominant source of variance is the difference between the two substrates. The corresponding loading plot highlights that this separation is mainly driven by variations in the G (∼1580 *cm* ^*–1*^) and 2D (∼2700 *cm* ^*–1*^) bands, consistent with substrate-induced differences in electronic and structural properties.

**Figure 7:**
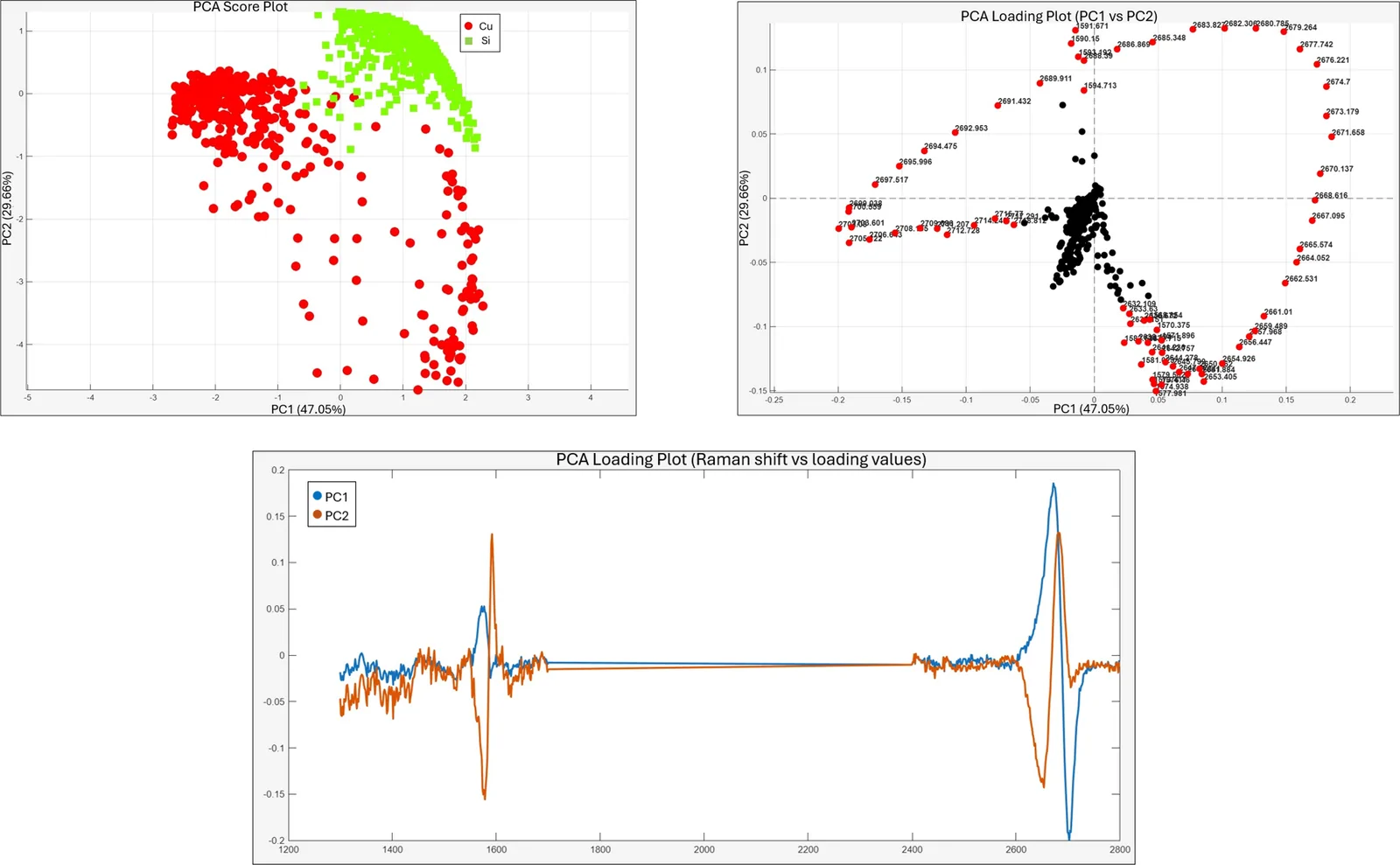
Principal component analysis (PCA) of the combined Raman data set acquired on graphene on Si and Cu. The score plot (PC1 vs PC2; upper-left panel). The corresponding loadings plots (reported in the upper-right and bottom panels) show the contribution of the different wavenumbers in the definition of the components. Each point in the loading plot corresponds to a Raman shift variable; points farther from the origin have a larger contribution to the corresponding principal components. Red points are used only as a visual guide to highlight the Raman-shift variables that appear farther from the origin in the loading space, while black points indicate the remaining variables. No quantitative threshold or independent color scale was applied for this graphical highlighting. In particular, the bottom panel reports the PC1 (blue) and PC2 (orange) loading values as a function of the Raman shift (cm^–1^). PCA performed separately on each data set does not show distinct clustering for graphene on Si (see Supplementary Figures S6–S7).

PCA performed on the individual data sets reveals that higher-order components (PC2 and PC3) do not introduce further separation (see Supplementary Figures S8–S11), confirming that Raman spectroscopy is primarily sensitive to average substrate-dependent effects, while intrasample variability remains secondary.

The spatial distribution of PCA scores provides further insight into the nature of this variability. As shown in Figure 8, PC1, PC2, and PC3 maps obtained from Raman measurements on both graphene on Si and graphene on Cu exhibit relatively smooth spatial variations, without strongly localized features. It is important to note that these maps do not represent direct physical quantities but, rather, the spatial distribution of spectral variations described by each principal component. In this case, the smooth behavior of PC1, PC2, and PC3 indicates that the underlying spectral variability is weakly structured in space, consistent with the limited dispersion observed in intradata set PCA (Supplementary Figures S6–S7).

**Figure 8:**
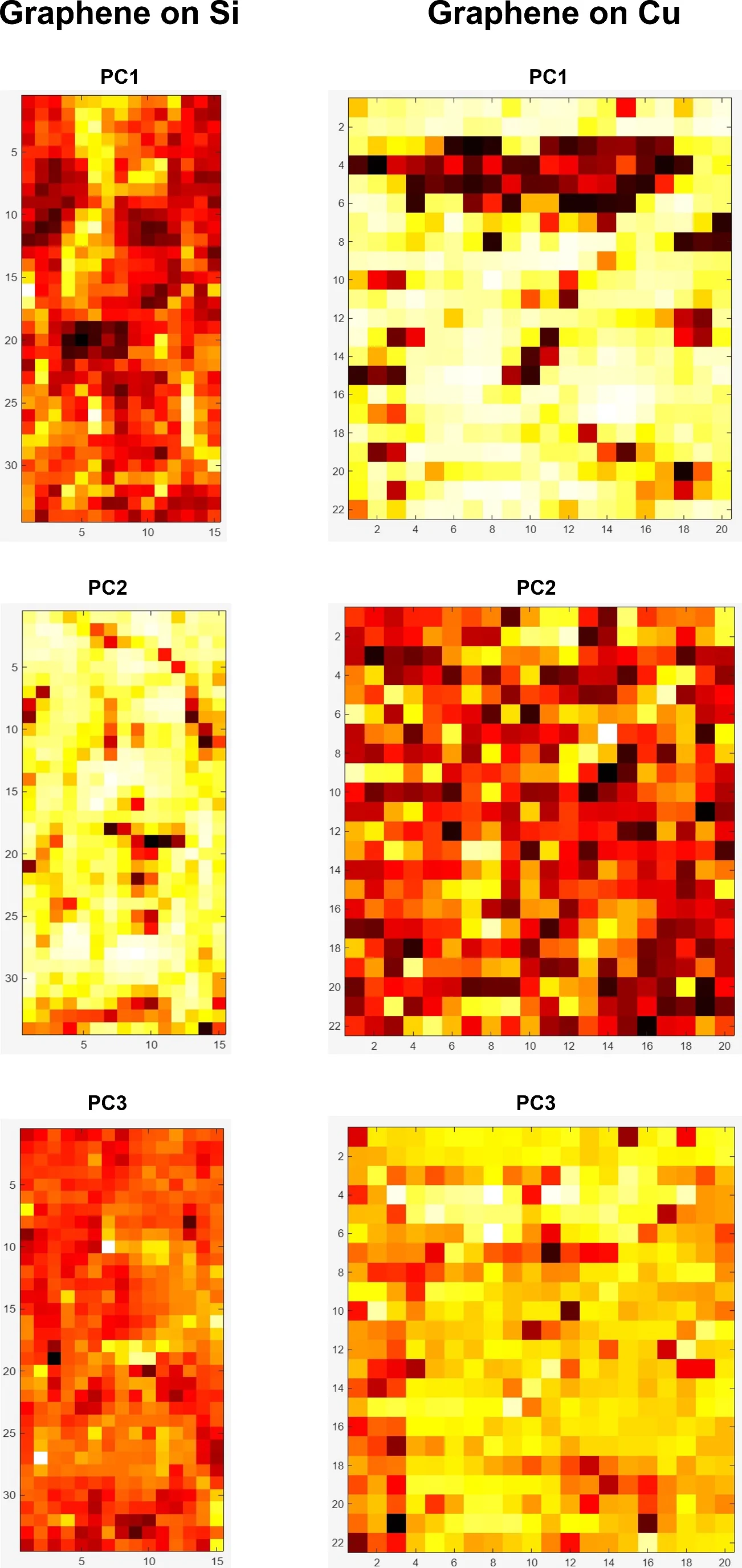
Spatial distribution of PCA scores (pixel size 1um × 1 um) obtained from Raman measurements on graphene on Si (left) and graphene on Cu (right). The maps are independently scaled for each principal component and data set; therefore, colors indicate relative score variations within the same map and should not be compared quantitatively between different maps, components, or samples.

#### 3.4.2 TERS Analysis

Different behaviors are observed in TERS data. PCA performed separately on graphene on Cu and graphene on Si already shows a broader dispersion of scores compared to Raman (see Supplementary Figures S12–S13). The loading plots suggest that, in addition to the G and 2D bands, defect-related spectral regions (∼1350 *cm* ^*–1*^) contribute more significantly to higher-order components. This indicates that TERS is sensitive to local variations in graphene properties even within the same substrate.

When the two TERS data sets are combined, PCA reveals a clear separation between graphene on Cu and Si along PC1 (Figure 9), similar to Raman. However, the distribution of the scores is significantly broader. The PC1 vs PC3 projection further highlights this behavior, showing that higher-order components capture substantial variability, particularly for graphene on Cu. The corresponding loading plot confirms that PC1 is still dominated by G and 2D contributions, whereas PC2 and PC3 include additional spectral features, suggesting multiple sources of variability.

**Figure 9:**
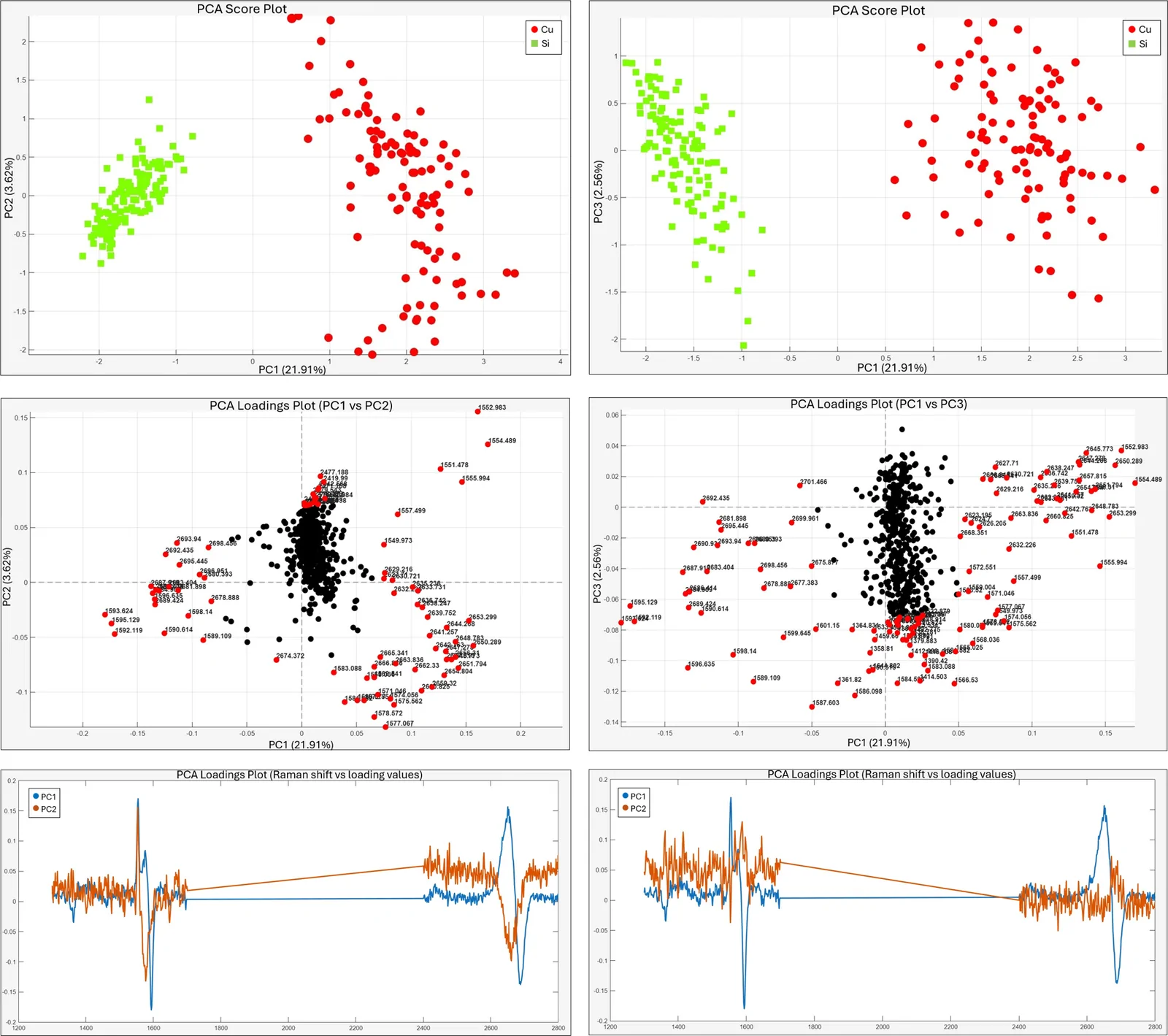
Principal component analysis (PCA) of the combined TERS data set acquired on graphene on Si and Cu. Score plots (PC1 vs PC2 and PC1 vs PC3, uppermost panels). The corresponding loadings plots are reported in the central panels. Each point in the loading plot corresponds to a Raman shift variable; points farther from the origin have a larger contribution to the corresponding principal components. Red points are used only as a visual guide to highlight the Raman-shift variables that appear farther from the origin in the loading space, while black points indicate the remaining variables. No quantitative threshold or independent color scale was applied for this graphical highlighting. The bottommost panels show the PC loading values as a function of the Raman shift (cm^–1^). Compared to Raman, the distribution is significantly broader, particularly for graphene on Cu, indicating increased variability.

Compared to Raman, TERS PCA therefore reveals both substrate-dependent differences (captured by PC1) and additional variability within each data set (captured by PC2 and PC3), which reflects local spectral fluctuations rather than distinct classes.

This interpretation is further supported by the spatial mapping of PCA scores. As shown in Figure 10, the PC1, PC2, and PC3 score maps derived from TERS measurements on graphene on Cu and graphene on Si reveal spatial variations that are more localized than those observed in the corresponding far-field Raman maps. In the Raman maps, adjacent pixels generally show similar colors and gradual transitions over relatively large areas, consistent with the larger probing volume and the spatial averaging inherent to far-field Raman spectroscopy. In contrast, the TERS score maps show more abrupt pixel-to-pixel variations and more confined regions of high contrast, indicating that the spectral variations described by the principal components are spatially structured at the nanoscale. The score maps were scaled independently for each principal component and data set to visualize the internal spatial distribution of the corresponding PCA scores. Therefore, the color scale should be interpreted only within each individual map: pixels with similar colors on the same map have similar PCA score values for that component, but colors should not be compared quantitatively between different maps, components, or samples. Moreover, these maps represent variations in the spectral response and should not be interpreted as direct quantitative maps of physical properties such as strain, doping, or defect density. The observed spatial patterns are therefore interpreted as evidence of nanoscale heterogeneity in the local spectroscopic response, which may arise from a combination of factors, including local strain, doping, defects, and tip–sample interaction effects. However, since corresponding SEM and AFM images of the bare substrates are not available, a possible contribution from the underlying substrate morphology cannot be excluded. This aspect is particularly relevant for graphene on polycrystalline Cu, where surface roughness, grains, grain boundaries, polishing marks, and possible oxide-related surface features may contribute to the observed morphology and locally influence strain, doping, and tip–sample coupling. For graphene transferred onto Si/SiO_2_, the thermally grown oxide provides a more defined substrate surface, although transfer-related wrinkles, residues, and local adhesion variations may still contribute to the observed spatial heterogeneity. In addition to these morphological effects, the possible influence of the substrate on the TERS enhancement mechanism must also be considered. In the case of graphene on Cu, the metallic substrate may contribute to a stronger and more spatially confined near-field response, possibly including a gap-mode-like contribution between the TiN tip and the Cu surface, although weaker than that typically obtained with noble-metal tips or substrates. This interpretation is consistent with the observation of the N_2_-related band around ∼ 2320 *cm* ^*–1*^ only for graphene on Cu, suggesting a stronger sensitivity to the local tip–sample environment and to substrate-assisted enhancement effects. In contrast, for graphene transferred onto Si/SiO_2_, the enhancement is expected to be mainly governed by the localized field at the tip apex, without a comparable metallic gap-mode contribution. These substrate-dependent morphological and enhancement-related effects may influence the apparent degree of nanoscale heterogeneity by affecting local strain and doping, enhancement efficiency, signal-to-noise ratio, and effective spatial averaging. Nevertheless, the main comparison between the two systems is not based on absolute TERS intensities but on normalized spectral profiles, peak positions, G-2D correlation trends, and PCA performed after preprocessing. In addition, the tip-out spectra are very weak for both substrates and almost negligible for graphene on Cu, indicating that the TERS response is dominated by the near-field contribution and that far-field averaging is limited. Therefore, while substrate morphology and substrate-dependent enhancement effects may contribute to the observed spectral contrast, the systematic variations in the G and 2D peak positions and the Raman/TERS comparison within each sample support the assignment of the observed heterogeneity to local strain- and doping-related variations in the graphene layer.

**Figure 10:**
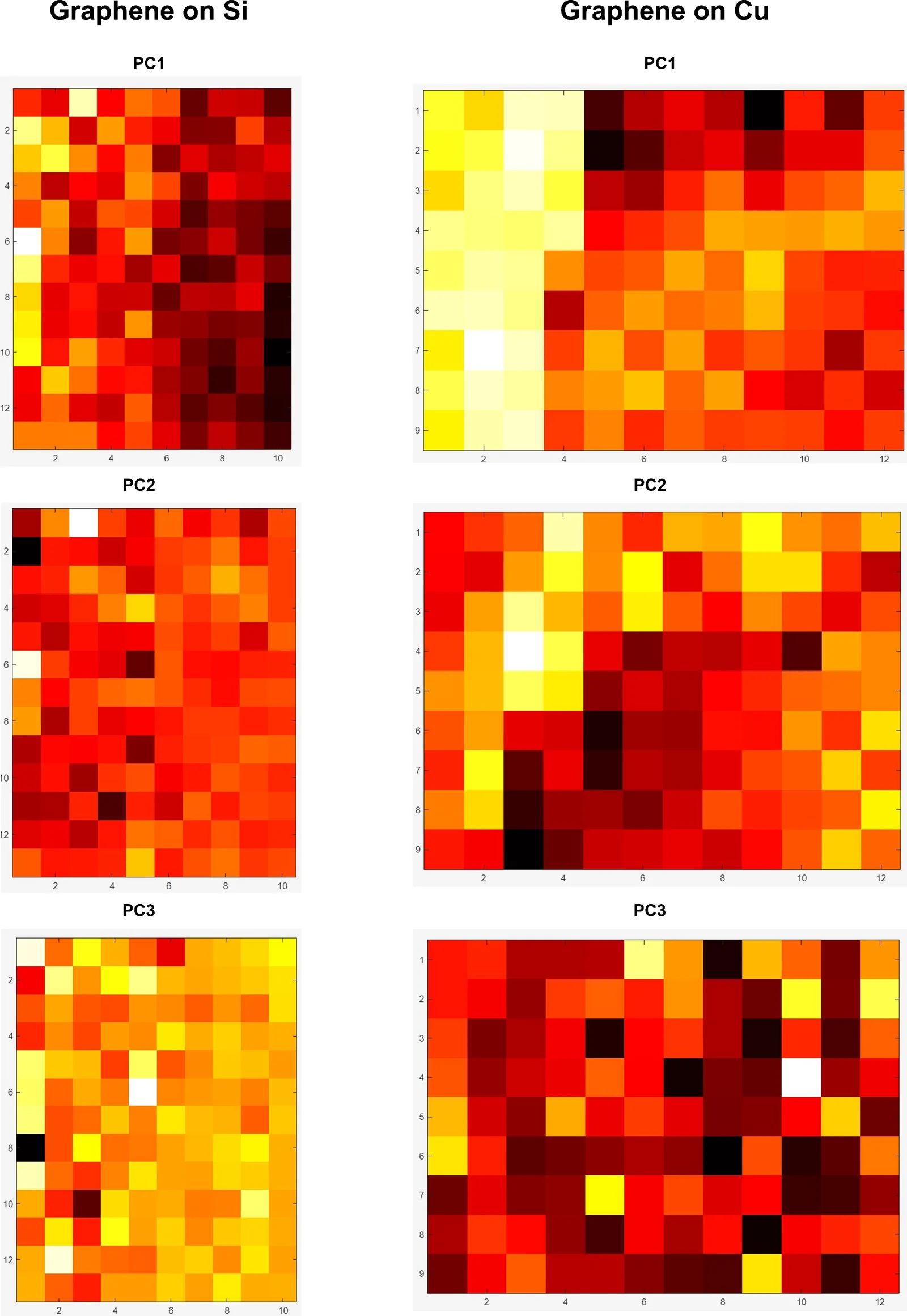
Spatial mapping of PCA scores (pixel sizes 75 nm × 75 nm for Si and 275 nm × 275 nm for Cu) derived from TERS measurements on graphene on Si (left) and graphene on Cu (right). The maps are independently scaled for each principal component and data set; therefore, colors indicate relative score variations within the same map and should not be compared quantitatively between different maps, components, or samples.

## 4 Conclusions

In this work, graphene grown on Cu and graphene transferred onto Si substrates were investigated by combining conventional Raman spectroscopy, tip-enhanced Raman spectroscopy (TERS), G-2D correlation analysis, and PCA. The aim was not only to compare the average Raman response of the two systems but also to determine how substrate-dependent strain- and doping-related effects are distributed from the microscale to the nanoscale.

Far-field Raman measurements revealed clear differences between the two substrates. Graphene on Cu showed a broader distribution of G and 2D peak positions, whereas graphene on Si exhibited a more compact spectral response. The G-2D correlation analysis indicated mixed strain- and doping-like contributions in both systems, with a more defined doping-related trend for graphene transferred onto Si. However, because of the larger probing volume, conventional Raman mainly provides an average description of the substrate-induced spectral response and only limited information on local heterogeneity.

TERS provided specific additional insight that these substrate-dependent spectral differences are not spatially uniform at the nanoscale. For graphene on Cu, the broader and more structured distribution observed in both the G-2D correlation plots and PCA space indicates a stronger local variability of the Raman response. This behavior is consistent with a nanoscale distribution of strain and charge transfer effects associated with the direct interaction between graphene and the metallic Cu substrate, together with local morphological and growth-related variations. For graphene transferred onto Si, the comparatively more compact TERS response suggests a more homogeneous spectral behavior, although local variations remain present and may be related to transfer-induced effects, local adhesion differences, residual surface contamination, and interaction with the SiO_2_ layer.

PCA further showed that the dominant substrate-related spectral contribution could be separated from additional local sources of variability. In particular, the TERS score maps displayed nanoscale spatial patterns that were not evident in the far-field Raman maps, confirming that part of the spectral variability is averaged out in the conventional Raman measurements. Therefore, the main contribution of TERS in this study is not only the established ability to acquire Raman information beyond the diffraction limit but also the identification of how strain- and doping-related spectral variations are locally distributed in graphene depending on the growth or transfer route and on the underlying substrate.

Overall, conventional Raman provides a reliable picture of the average substrate-induced response of graphene, whereas TERS reveals the nanoscale heterogeneity hidden within the average response. The combined use of far-field Raman, TERS, G-2D correlation analysis, and PCA therefore offers a useful framework for investigating graphene and other two-dimensional materials in which local strain, doping, defects, adhesion, and substrate interactions can strongly influence the macroscopic material response.

## Supplementary Material



## Notes

Comparison of far-field Raman spectra acquired from graphene on Cu and Si; tip-in and tip-out Raman spectra acquired from graphene on Cu and Si; comparison of TERS and far-field Raman spectra; PCA score and loading plots obtained from Raman and TERS data sets for graphene on Cu and Si using different principal-component combinations (PDF)

## Notes

During the preparation of this work, the author(s) used generative artificial intelligence (AI) tools to support language refinement, stylistic revision. All AI-assisted content was carefully reviewed, edited, and validated by the author(s), who take full responsibility for the accuracy, integrity, and final content of the published article.

## Abbreviations

TERS: Tip Enhanced Raman Spectroscopy
PCA: Principal Component Analysis
CVD: Chemical Vapor Deposition
SEM: Scanning Electron Microscopy
EDX: Energy Dispersive X-ray Spectroscopy
SDD: Silicon Drift Detector
AFM: Atomic Force Microscopy
PC: Principal Component
