# Supplementary material for: Nanoscale Disentangling of Strain and Doping in Monolayer Graphene Grown on Cu-Based Growth Template and Transferred onto Si Substrate by Combining TERS, Raman Correlation Analysis, and PCA
Source: ACS Omega. 2026 Aug 6;11(34):51663–76. doi: 10.1021/acsomega.6c05883 (PMC13625541; doi:10.1021/acsomega.6c05883)
Supplement: Supplementary file 1 [file ao6c05883_si_001.pdf]

Nanoscale Disentangling of Strain and Doping in Monolayer Graphene Grown on Cu-Based Growth Template and Transferred onto Si Substrate by Combining TERS, Raman Correlation Analysis, and PCA

*Anacleto Proietti 1, †, *, Luca, Buccini 1, †, Ginevra Rossetti 2, *, Livia Angeloni 1, Pierfrancesco Atanasio 1, Simone Bandini 1, Isabella Chiarotto 1, Roberto Colarelli 1, Federico Marini 2, Francesco Mura 1,3, Daniele Passeri 1,3, Gianluca Sbardella 1, Elena Tomaselli 1, Angelica Accorinti 4, Corrado Di Conzo 4, Alba Centeno 5, Amaia Zurutuza 5, Marco Rossi 1,3*

1 Department of Basic and Applied Sciences for Engineering, Sapienza University of Rome, Via A. Scarpa 16, 00161 Rome, Italy

2 Department of Chemistry, Sapienza University of Rome, Piazzale Aldo Moro 5, 00185 Rome, Italy

3 Research Center for Nanotechnology applied to Engineering of Sapienza University of Rome, Piazzale Aldo Moro 5, 00185 Rome, Italy

4 Department of Applied Science and Technology (DISAT), Polytechnic University of Turin, Corso Castelfidardo 39, 10129 Turin, Italy

5 Graphenea S.A, Paseo Mikeletegi 83, 20009 San Sebastián, Spain

† These authors contributed equally to this work and share first authorship.

* Corresponding authors: Anacleto Proietti1 (anacleto.proietti@uniroma1.it); Ginevra Rossetti2 (ginevra.rossetti@gmail.com)

Keywords: Tip-enhanced Raman Spectroscopy, Graphene, Strain and Doping, Principal Component Analysis, Two-Dimensional Materials

Author contributions:

A.P. and L.B. contributed equally to this work and share first authorship. Conceptualization: A.P., L.B., and M.R. Methodology: A.P., L.B., G.R., F.M. (Marini), D.P., and M.R. Investigation: A.P., L.B., F.M.(Mura), P.A., S.B., I.C., and A.A. Formal analysis: A.P., L.B., F.M. (Marini), and G.R. Data curation: A.P., L.B., E.T., A.A., and G.R. Visualization: A.P., L.B., C.D.C., and F.M. (Mura). Validation: I.C., G.S., R.C., E.T., A.A., and C.D.C. Resources: M.R., A.C. and A.Z. Writing – original draft: A.P. and L.B. Writing – review & editing: all authors. Supervision: L.A., D.P., and M.R. Funding acquisition: M.R.

Funding Sources: This work was supported by the project iENTRANCE@ENL: Infrastructure for Energy TRAnsition aNd Circular Economy @ EuroNanoLab (Project code: IR0000027; CUP: B33C22000710006), funded by the European Union – NextGenerationEU under the National Recovery and Resilience Plan (NRRP), Mission 4 "Education and Research", Component 2 "From Research to Business", Investment 3.1 "Fund for the realization of an integrated system of research and innovation infrastructures", Call No. 3264 of 28 December 2021 issued by the Italian Ministry of University and Research, Award Decree No. 128 of 21 June 2022.

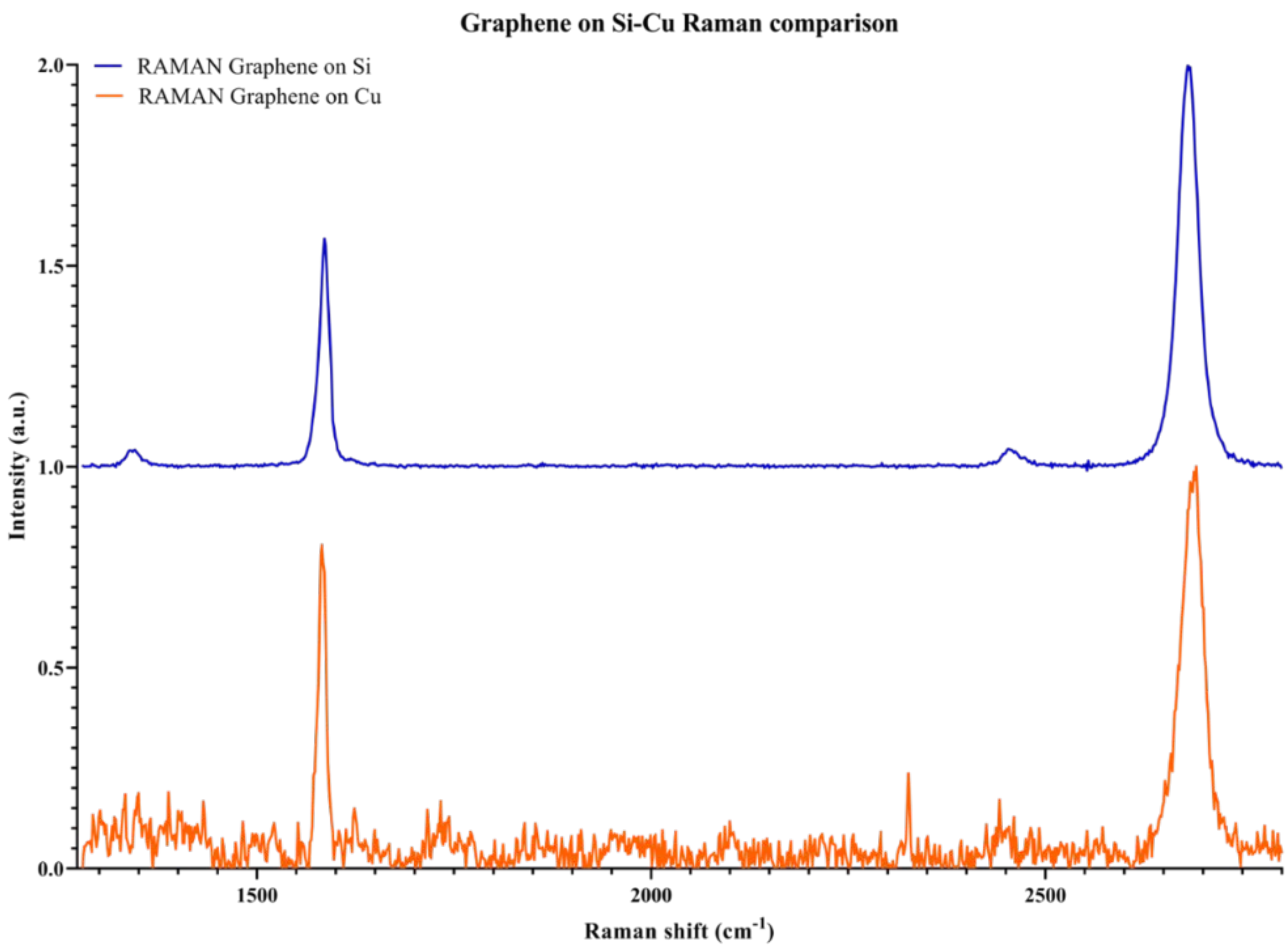

Figure S1. Comparison of far-field Raman spectra acquired on the investigated graphene samples. The spectra, vertically offset for clarity, shows the characteristic G and 2D bands, with differences in intensity, signal-to-noise ratio, and background contributions between the two measurements (PNG).

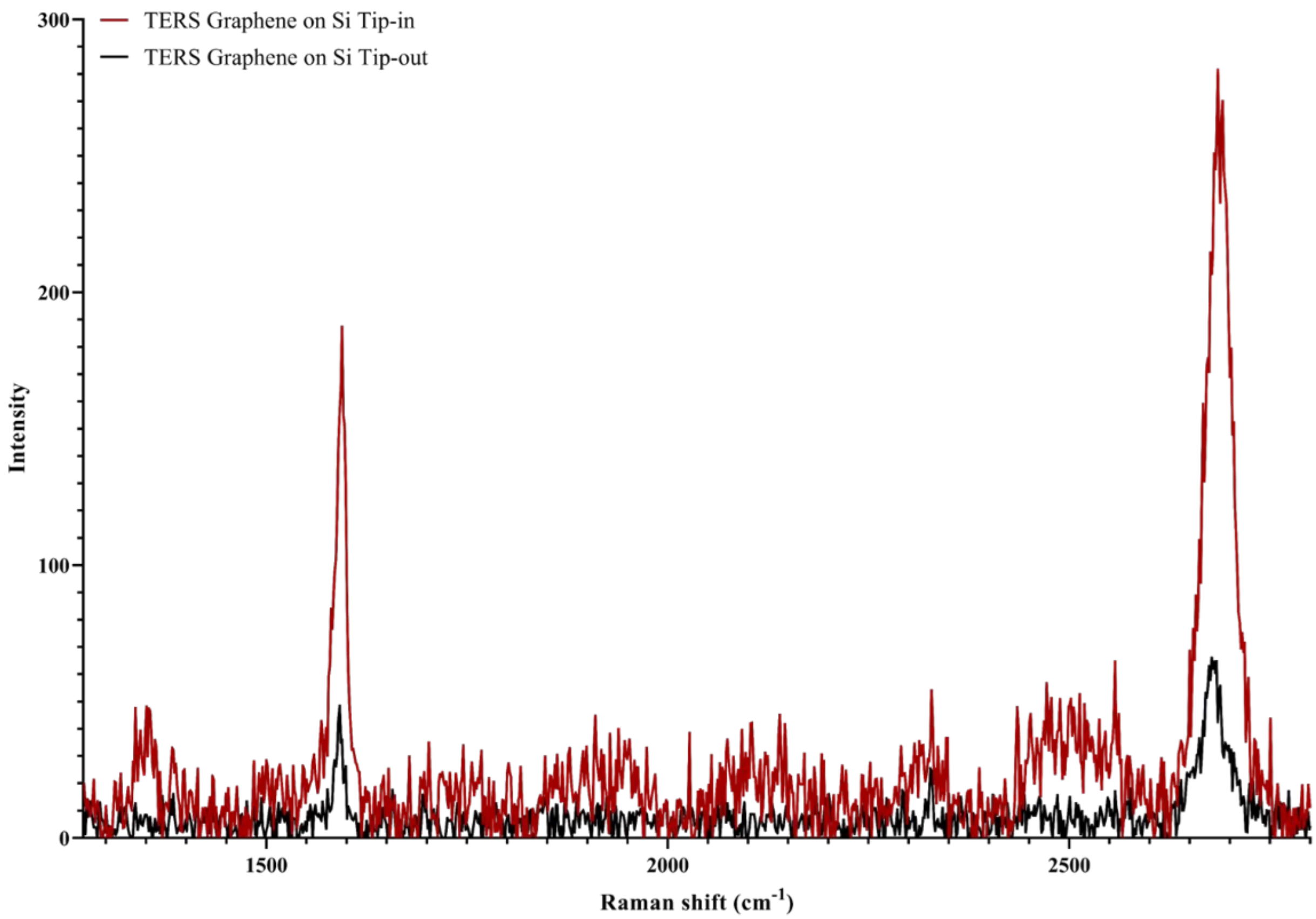

Figure S2. Tip-in and tip-out Raman spectra acquired on graphene on Si. The spectra are vertically offset for clarity (PNG).

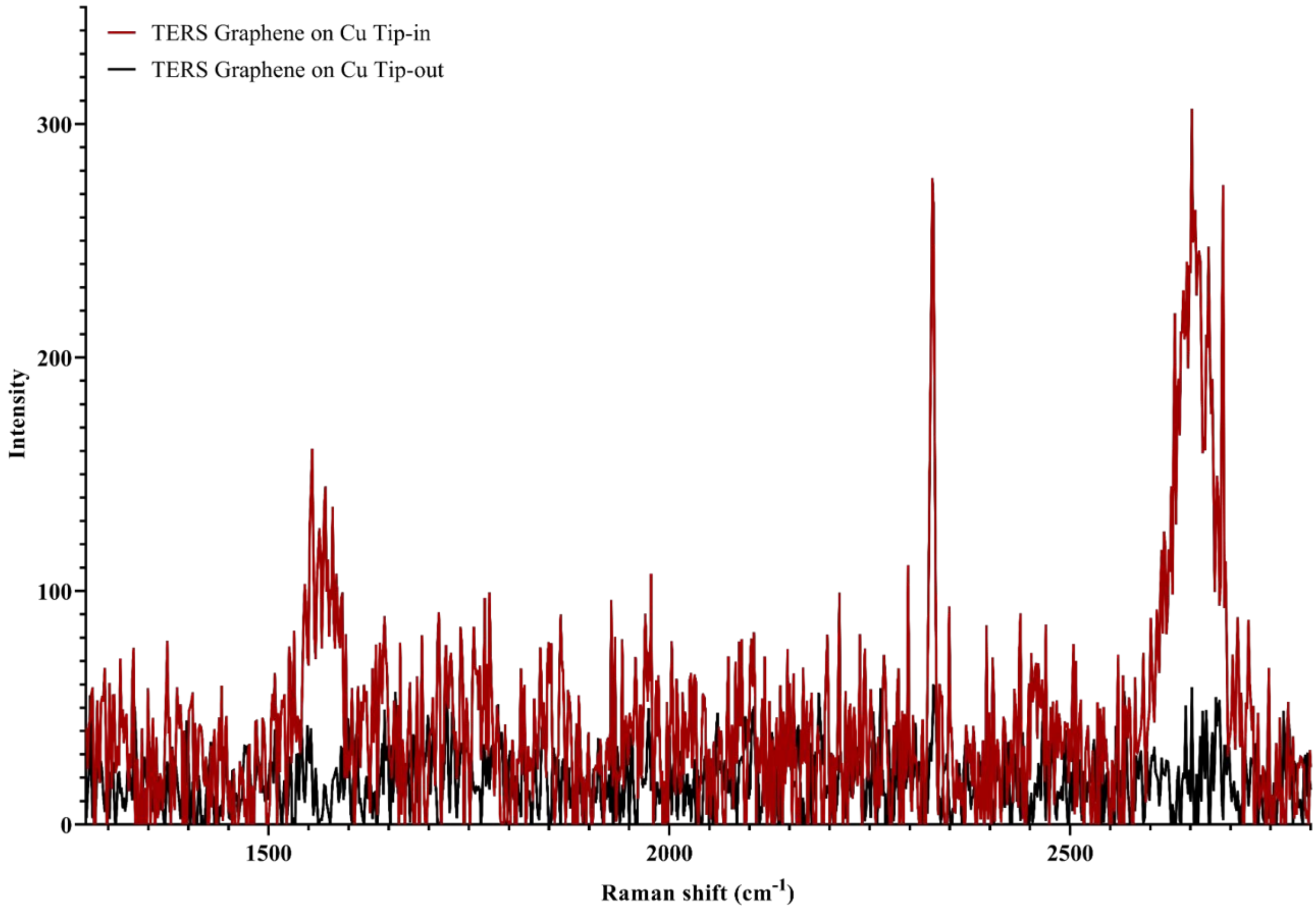

Figure S3. Tip-in and tip-out Raman spectra acquired on graphene on Cu. The spectra are vertically offset for clarity (PNG).

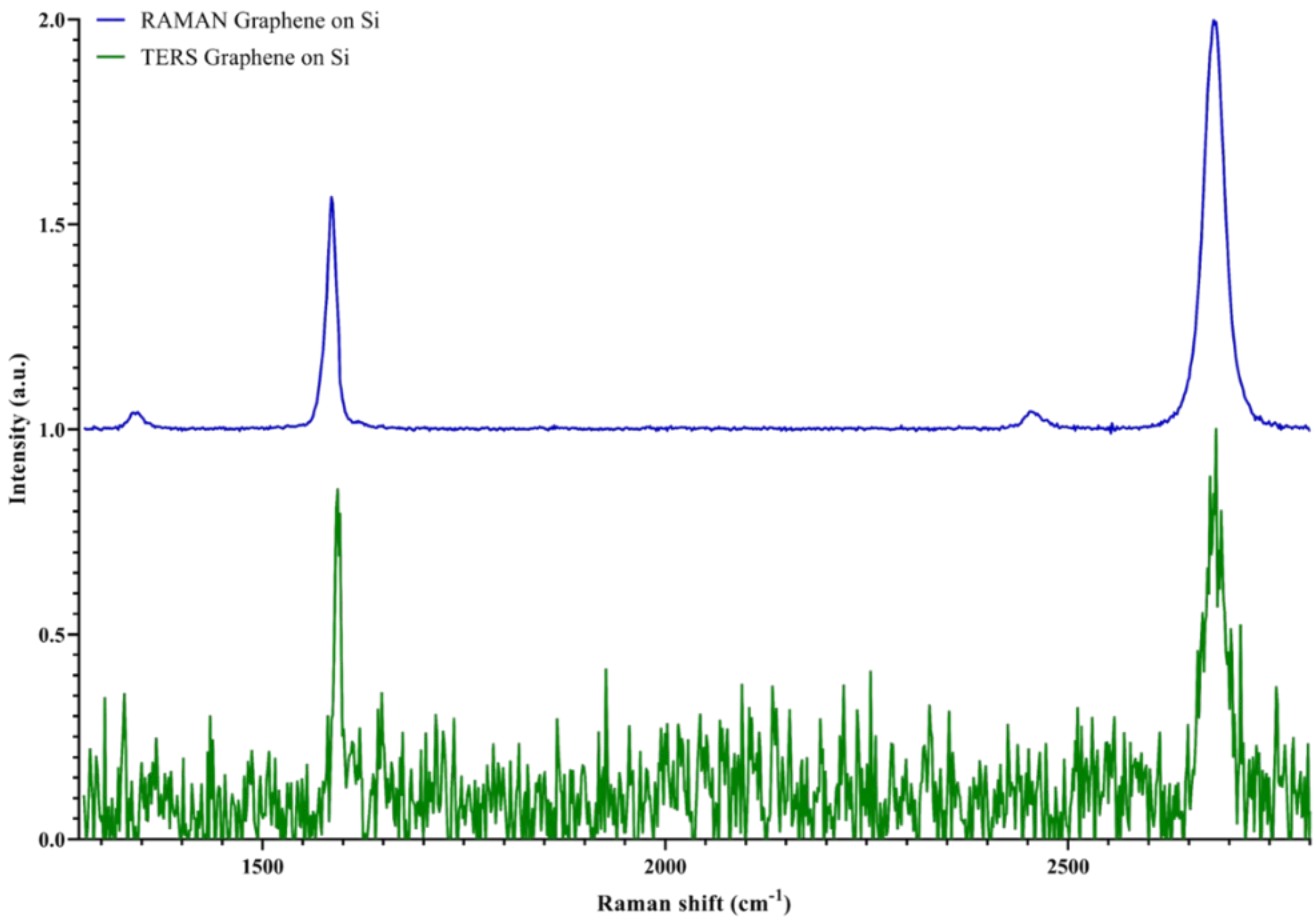

Figure S4. Comparison between TERS (tip-in) and far-field Raman spectra for graphene on Si. The spectra are vertically offset for clarity (PNG).

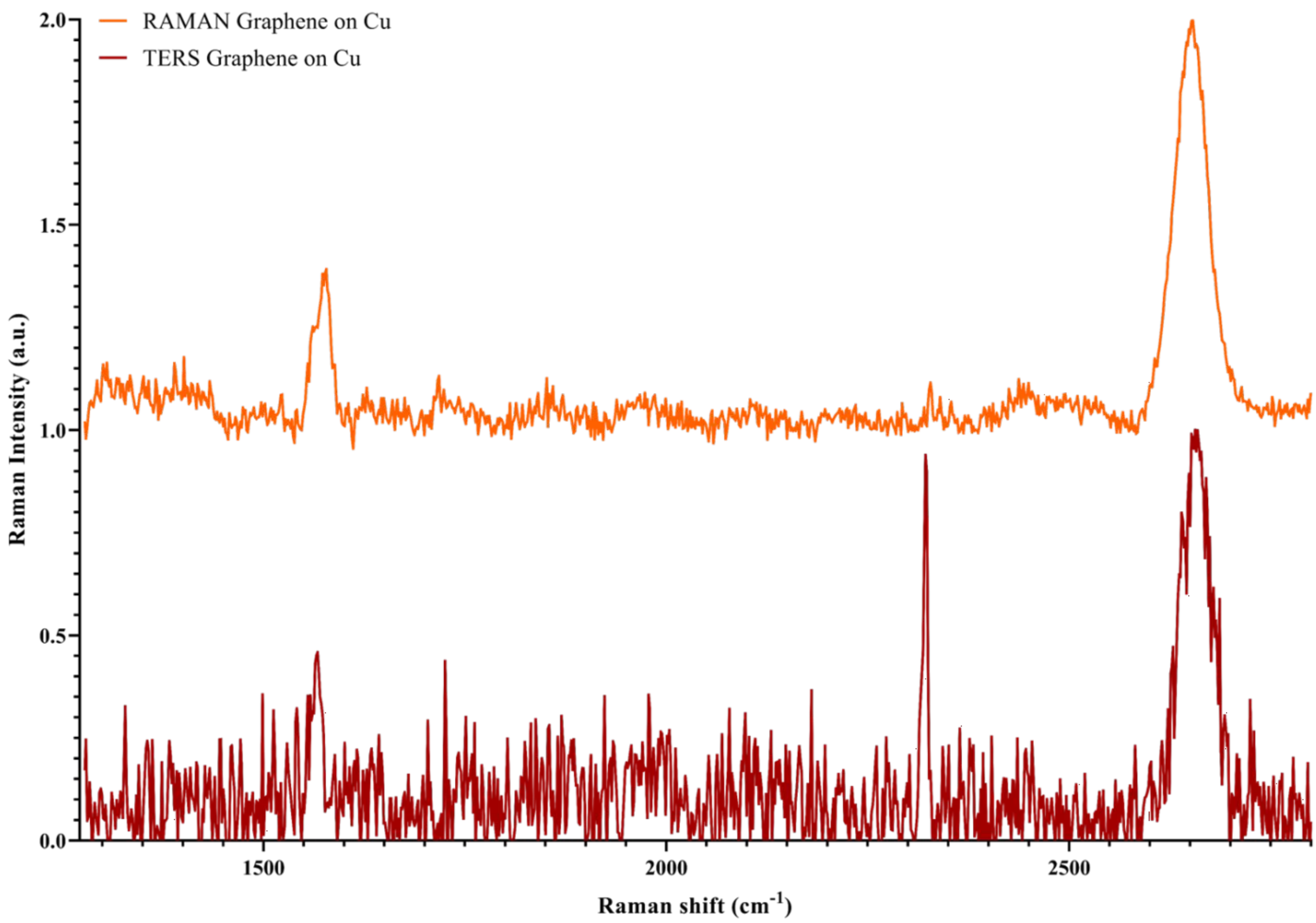

Figure S5. Comparison between TERS (tip-in) and far-field Raman spectra for graphene on Cu. The spectra are vertically offset for clarity (PNG).

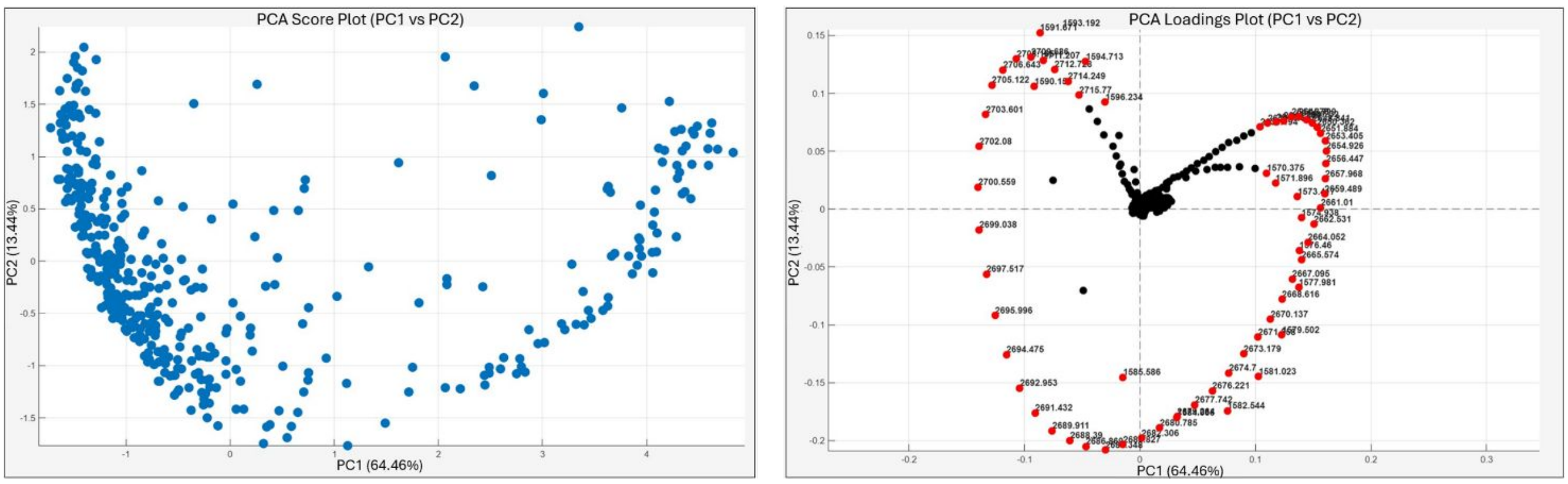

Figure S6. PCA score and loadings plot (PC1 vs PC2) obtained from Raman spectra acquired on graphene on Cu. *Each point in the loading plot corresponds to a Raman shift variable, points farther from the origin have a larger contribution to the corresponding principal components. Red points are used only as a visual guide to highlight the Raman-shift variables that appear farther from the origin in the loading space, while black points indicate the remaining variables. No quantitative threshold or independent color scale was applied for this graphical highlighting (PNG).*

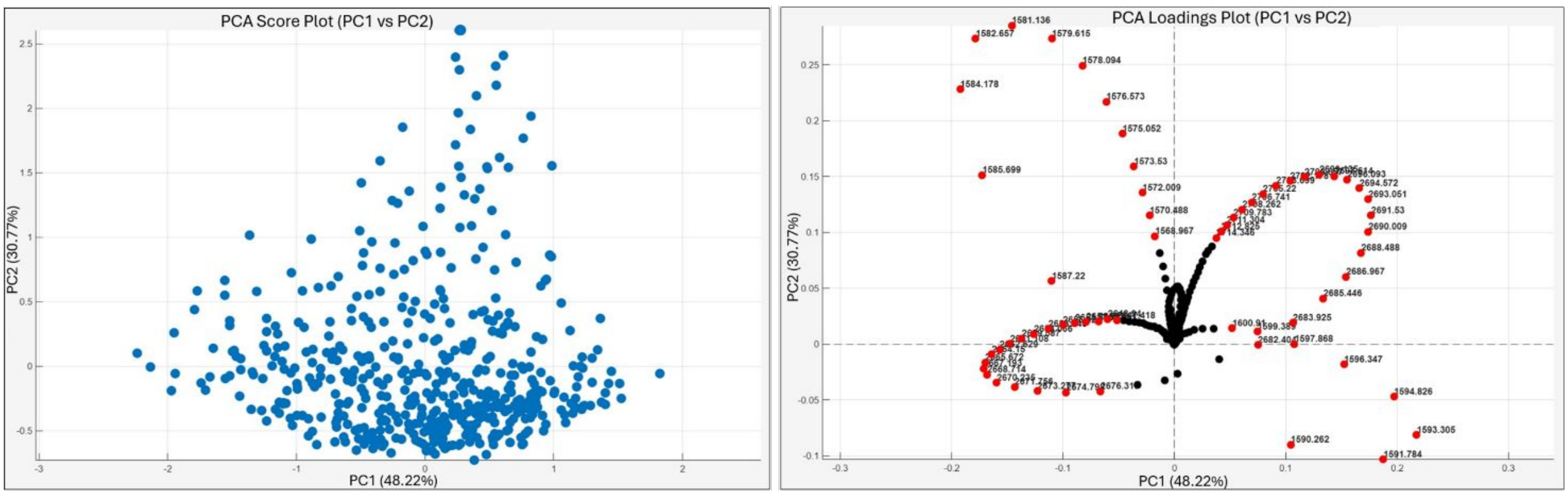

Figure S7. PCA score and loadings plot (PC1 vs PC2) obtained from Raman spectra acquired on graphene on Si. *Each point in the loading plot corresponds to a Raman shift variable, points farther from the origin have a larger contribution to the corresponding principal components. Red points are used only as a visual guide to highlight the Raman-shift variables that appear farther from the origin in the loading space, while black points indicate the remaining variables. No quantitative threshold or independent color scale was applied for this graphical highlighting (PNG).*

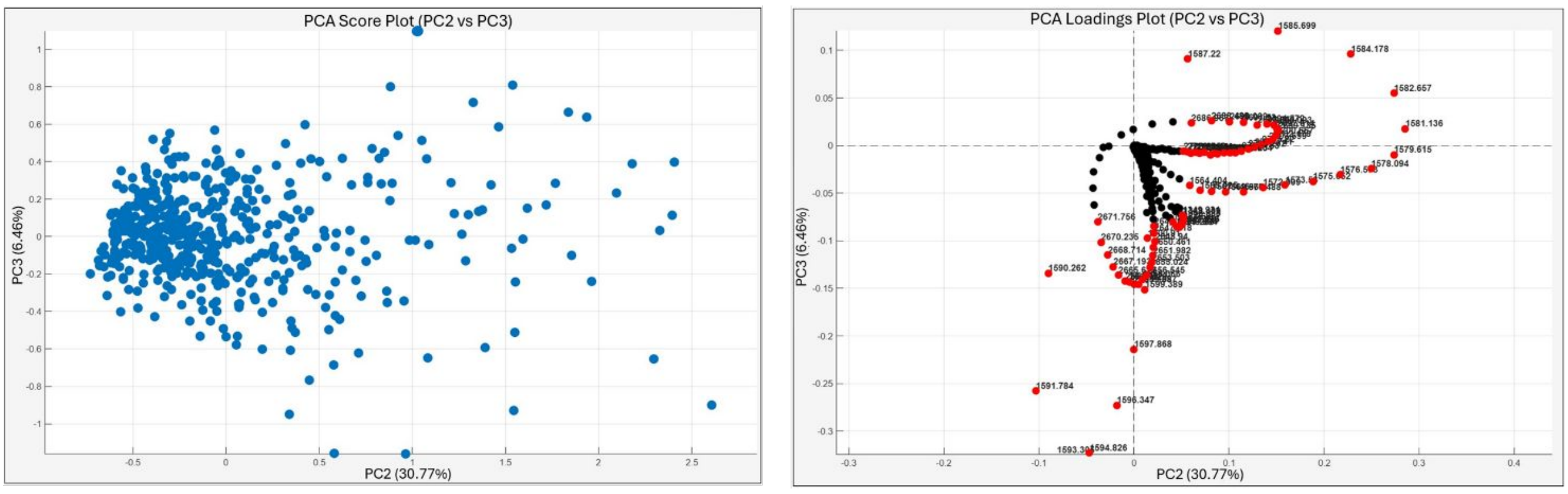

Figure S8. PCA score and loadings plot (PC2 vs PC3) obtained from Raman spectra acquired on graphene on Si. *Each point in the loading plot corresponds to a Raman shift variable, points farther from the origin have a larger contribution to the corresponding principal components. Red points are used only as a visual guide to highlight the Raman-shift variables that appear farther from the origin in the loading space, while black points indicate the remaining variables. No quantitative threshold or independent color scale was applied for this graphical highlighting (PNG).*

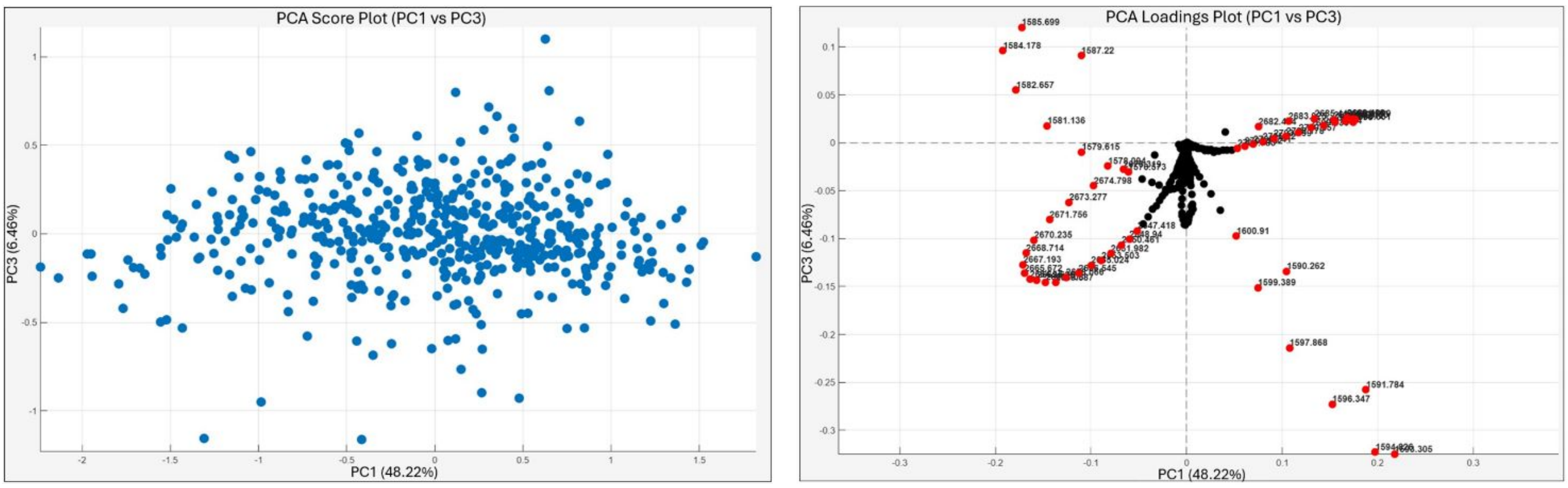

Figure S9. PCA score and loadings plot (PC1 vs PC3) obtained from Raman spectra acquired on graphene on Si. *Each point in the loading plot corresponds to a Raman shift variable, points farther from the origin have a larger contribution to the corresponding principal components. Red points are used only as a visual guide to highlight the Raman-shift variables that appear farther from the origin in the loading space, while black points indicate the remaining variables. No quantitative threshold or independent color scale was applied for this graphical highlighting (PNG).*

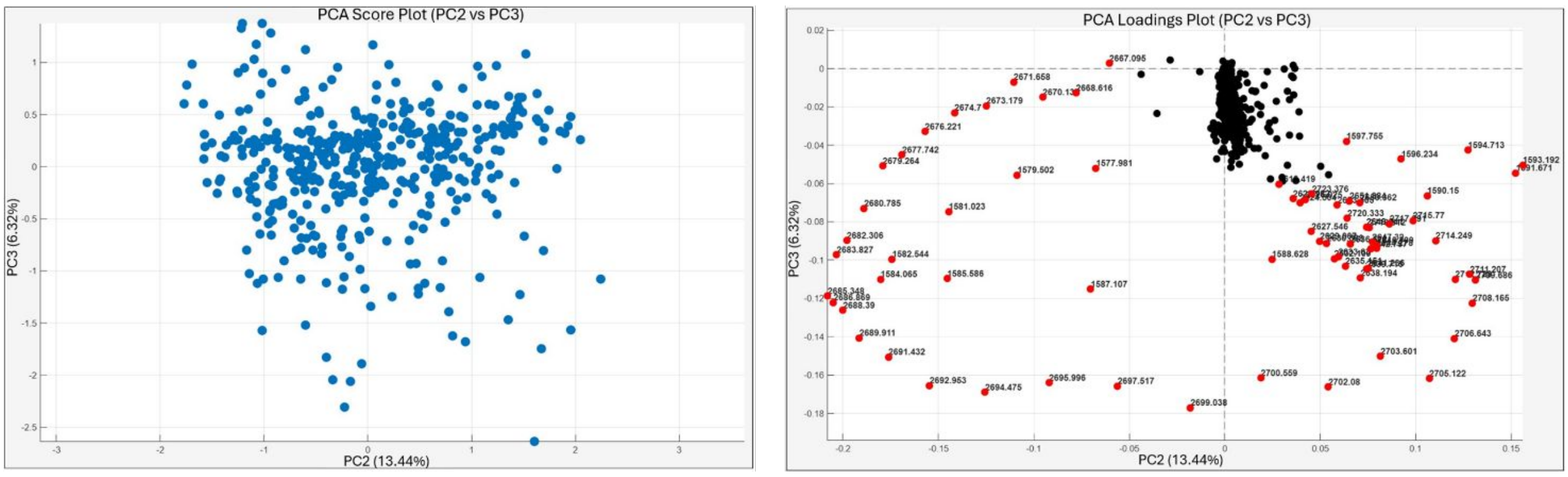

Figure S10. PCA score and loadings plot (PC2 vs PC3) obtained from Raman spectra acquired on graphene on Cu. *Each point in the loading plot corresponds to a Raman shift variable, points farther from the origin have a larger contribution to the corresponding principal components. Red points are used only as a visual guide to highlight the Raman-shift variables that appear farther from the origin in the loading space, while black points indicate the remaining variables. No quantitative threshold or independent color scale was applied for this graphical highlighting (PNG).*

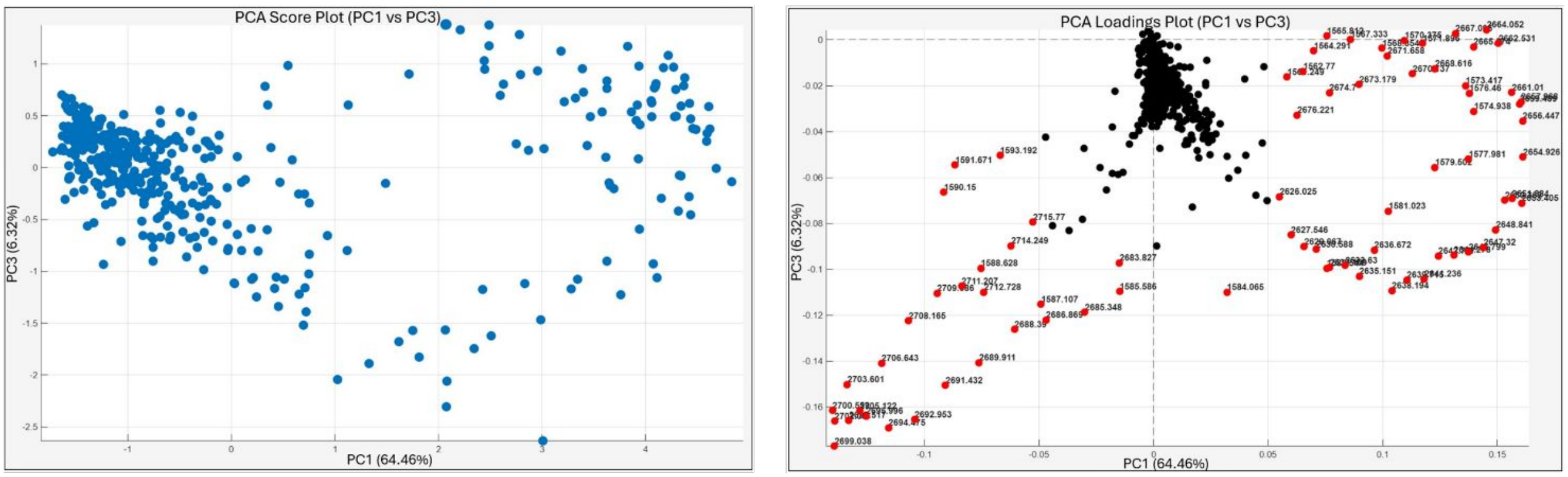

Figure S11. PCA score and loadings plot (PC1 vs PC3) obtained from Raman spectra acquired on graphene on Cu. *Each point in the loading plot corresponds to a Raman shift variable, points farther from the origin have a larger contribution to the corresponding principal components. Red points are used only as a visual guide to highlight the Raman-shift variables that appear farther from the origin in the loading space, while black points indicate the remaining variables. No quantitative threshold or independent color scale was applied for this graphical highlighting (PNG).*

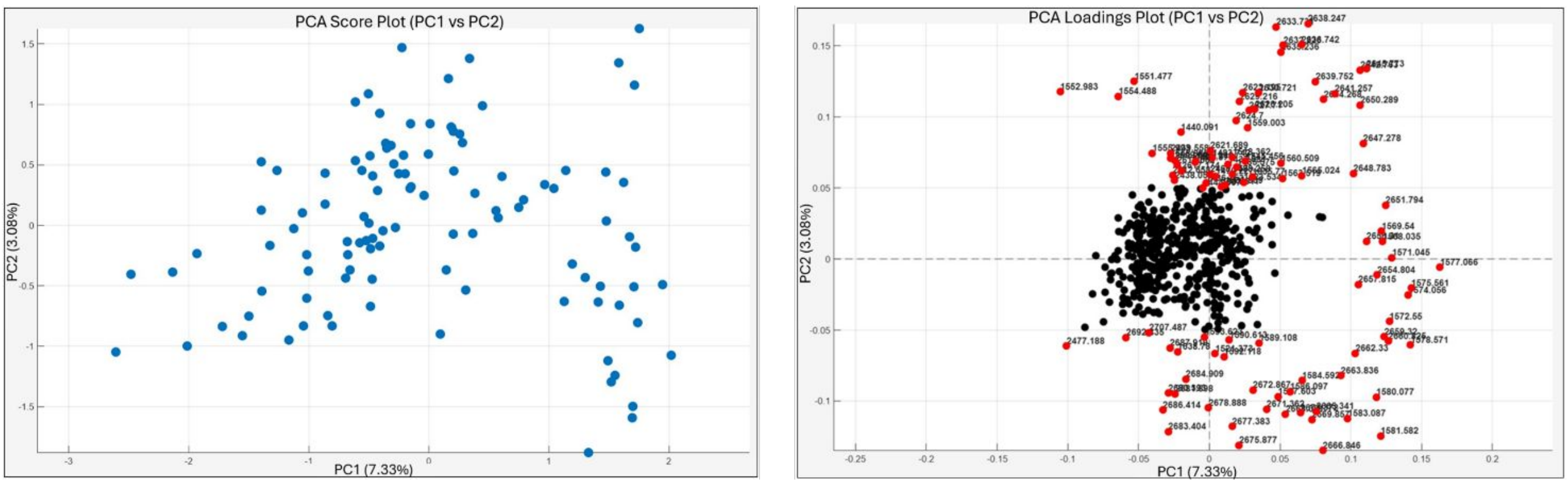

Figure S12. PCA score and loadings plot (PC1 vs PC2) obtained from TERS spectra acquired on graphene on Cu. *Each point in the loading plot corresponds to a Raman shift variable, points farther from the origin have a larger contribution to the corresponding principal components. Red points are used only as a visual guide to highlight the Raman-shift variables that appear farther from the origin in the loading space, while black points indicate the remaining variables. No quantitative threshold or independent color scale was applied for this graphical highlighting (PNG).*

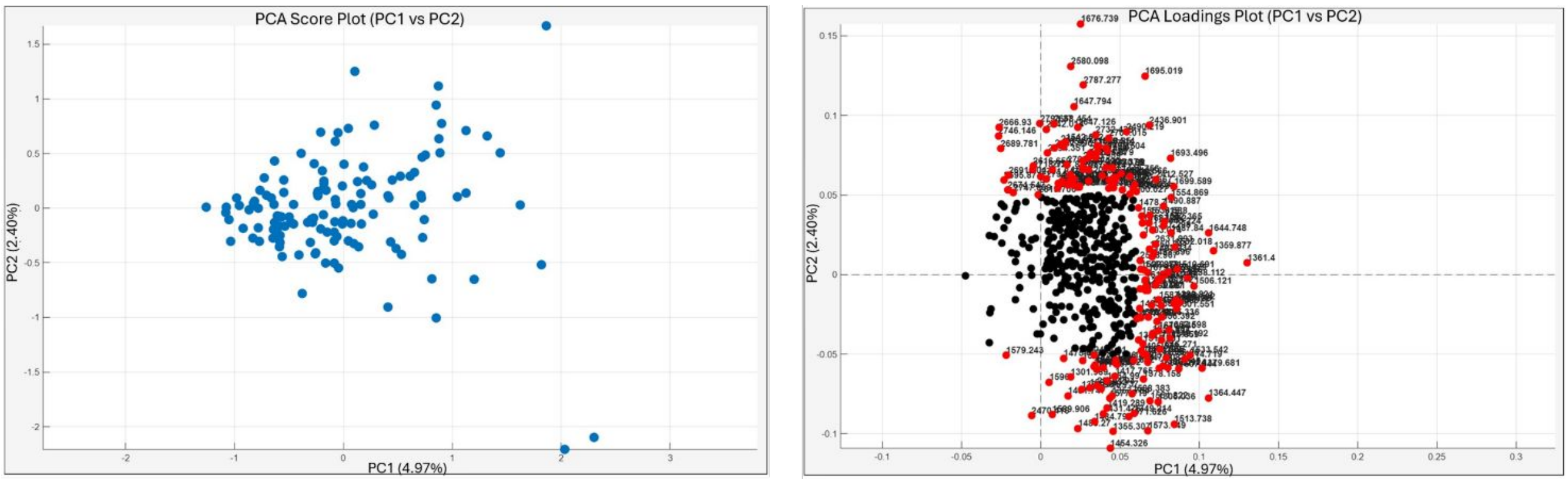

Figure S13. PCA score and loadings plot (PC1 vs PC2) obtained from TERS spectra acquired on graphene on Si. *Each point in the loading plot corresponds to a Raman shift variable, points farther from the origin have a larger contribution to the corresponding principal components. Red points are used only as a visual guide to highlight the Raman-shift variables that appear farther from the origin in the loading space, while black points indicate the remaining variables. No quantitative threshold or independent color scale was applied for this graphical highlighting (PNG).*
